# Admission blood-interstitial glucose gap shows metabolic and prognostic associations in mechanically ventilated patients

**DOI:** 10.1016/j.isci.2026.118093

**Published:** 2026-11-02

**Authors:** Zhibo Zhang, Tao Gao, Xinran Liang, Sijia Dai, Xiaohui Liang, Yun Xu, Ming Chen, Xiang Ji, Shi Chen, Jianfeng Duan, Gang Wang, Wenkui Yu

**Affiliations:** 1 Department of Critical Care Medicine, Nanjing Drum Tower Hospital, Drum Tower Clinical College, Nanjing University of Chinese Medicine, Nanjing 210008, China; 2 Department of Critical Care Medicine, Nanjing Drum Tower Hospital, Affiliated Hospital of Medical School, Nanjing University, Nanjing 210008, China; 3 Department of Emergency, The Affiliated Taizhou People’s Hospital of Nanjing Medical University, Taizhou 225300, China

**Keywords:** blood-interstitial glucose gap, insulin resistance, metabolic monitoring, metabolic subtypes, critically ill patients

## Abstract

Glucose concentration differences across vascular and interstitial compartments may provide complementary insights into metabolic status during critical illness. In this prospective observational study, we obtained synchronized arterial, venous, and interstitial glucose measurements from 140 mechanically ventilated patients. The arterial-venous glucose gap (Gap_A-V_) demonstrated greater discrimination for three-month all-cause mortality than the arterial-interstitial glucose gap (Gap_A-I_), with areas under the curve of 0.863 and 0.708, respectively. In contrast, Gap_A-I_ was strongly associated with insulin resistance (HOMA-IR, *r* = −0.85) and resting energy expenditure measured by indirect calorimetry (*r* = 0.79). Combining Gap_A-I_ with lactate identified four exploratory metabolic phenotypes with distinct metabolic characteristics and survival outcomes. These findings suggest that glucose gaps capture complementary prognostic and metabolic information, warranting further validation in independent cohorts.

## Highlights

• Admission glucose gaps reflect early metabolic status in ventilated patients
• Gap_A-V_ predicts mortality effectively, while Gap_A-I_ indicates insulin resistance
• Lactate and Gap_A-I_ stratification reveals four distinct metabolic phenotypes
• Uptake- and Dual-Defective phenotypes showed similarly low survival rates

## Graphical abstract

**Figure:**
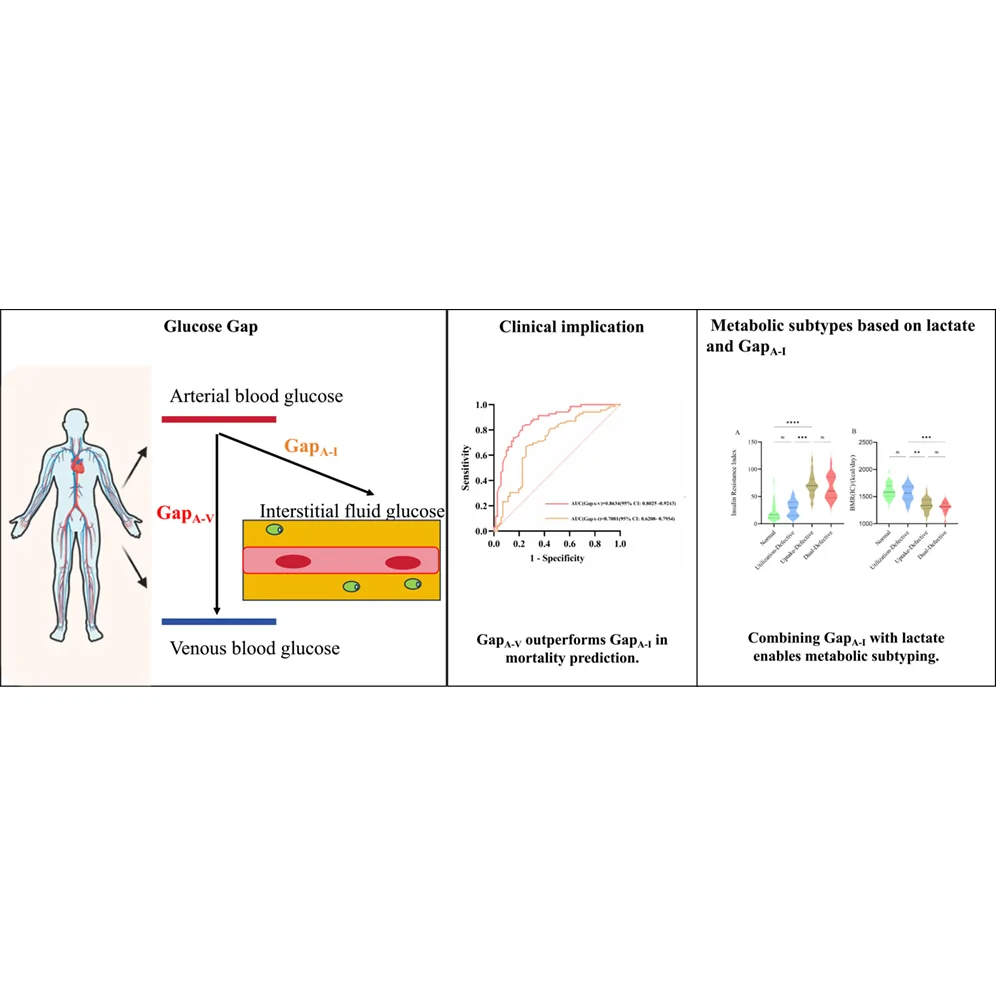

## Teaser

Health sciences

## Introduction

Metabolic monitoring serves as a critical component of nutritional therapy in critically ill patients.^1^^,^^2^ If nutritional support is not guided by accurate metabolic assessment, energy imbalance may occur, potentially exacerbating multiple organ dysfunction syndrome (MODS) and increasing mortality risk.^3^^,^^4^ Therefore, real-time and precise metabolic monitoring is essential for optimizing nutritional support and improving clinical outcomes in this vulnerable population.

Several methods are currently employed for metabolic monitoring in critical care. Predictive equations, which estimate energy expenditure based on static parameters such as body weight, height, and age, represent the most widely used approach in clinical practice.^5^ However, unlike metabolically stable patients, critically ill individuals frequently experience profound and rapid metabolic fluctuations. Relying solely on static parameters to estimate energy expenditure while ignoring dynamic metabolic status, physiological changes, and therapeutic interventions inevitably leads to inaccurate assessments.^6^ Indirect Calorimetry (IC), which calculates energy expenditure by measuring oxygen consumption and carbon dioxide production, is considered the gold standard for metabolic assessment in critically ill patients.^7^ Nevertheless, conventional IC has several limitations: it requires metabolic steady-state conditions; each measurement is time-consuming (15–30 min), making it unsuitable for capturing rapid metabolic changes^8^^,^^9^; and it may be inaccurate under special circumstances, such as during high-frequency ventilation or ECMO support, because of altered gas exchange pathways.^10^^,^^11^ These limitations highlight the urgent need to explore novel theories and technologies for metabolic monitoring.

Accurate substrate metabolism monitoring forms the foundation for determining actual energy expenditure.^12^ Glucose metabolism plays a pivotal role in substrate metabolism during the acute phase of critical illness.^13^ Research indicates that under stress conditions, downregulation of glucose transporter function leads to impaired peripheral glucose uptake,^14^ while mitochondrial dysfunction significantly compromises oxidative glucose metabolism by impairing pyruvate dehydrogenase complex activity.^15^ These dual-pathway disturbances in glucose uptake and utilization inevitably cause abnormal concentrations of glucose and its metabolites in body fluids (such as blood and interstitial fluid), manifesting as hyper/hypoglycemia and hyper/hypolactatemia.^16^^,^^17^ Physiologically, the arterial end of the microcirculation delivers glucose to tissues, cells take up and metabolize glucose from the interstitial fluid, and the venous end recovers metabolites and unused glucose.^18^ In critically ill patients, stress-induced microcirculatory impairment significantly alters glucose distribution. Recent evidence clearly demonstrates that predominant capillary leakage leads to glucose extravasation, significantly reducing the arterial-interstitial glucose concentration difference, while predominant blood flow stasis causes impaired glucose delivery, resulting in an abnormal increase in this gradient—both opposite patterns predict poor prognosis.^19^ Based on this physiological evidence, we hypothesize that glucose metabolic disturbances in critically ill patients are reflected not only in absolute blood glucose levels but, more crucially, in the glucose concentration differences along the arterial blood-interstitial fluid-venous blood microcirculatory pathway. Specifically, our formal hypothesis is that the glucose concentration gaps (Gap_A-V_ and Gap_A-I_) may reflect microcirculatory perfusion and cellular metabolic status. Furthermore, we hypothesize that narrowed glucose gaps may be associated with worsened organ dysfunction and may have prognostic relevance for poor clinical outcomes, particularly mortality, in critically ill patients.

Accordingly, this study aims to investigate the association between differences in glucose concentrations—arterial blood glucose (G_A_), interstitial fluid glucose (G_I_), and venous blood glucose (G_V_)—and resting energy expenditure, organ function, and clinical outcomes in critically ill patients. We anticipate that evaluating the clinical utility of this novel metric may contribute to improved metabolic monitoring in critically ill patients.

## Results

In this prospective observational study, 140 mechanically ventilated critically ill patients were included in the final analysis. For each patient, arterial blood glucose (G_A_) and venous blood glucose (G_V_) were determined by point-of-care blood gas analysis, whereas interstitial fluid glucose (G_I_) was recorded using a subcutaneous continuous glucose monitoring sensor implanted in the left lower abdomen (see STAR Methods). All three glucose measurements were obtained synchronously at a predefined single time point (T0), defined as 6 h after ICU admission to allow for sensor equilibration and initial patient stabilization. Based on these synchronized measurements, the arterial-venous glucose gap (*Gap*_*A*-*V*_ = *G*_*A*_-*G*_*V*_) and the arterial-interstitial glucose gap (${{Gap}_{A-I}=G_{A}-G}_{I}$) were calculated. We then sequentially examined the distributions of the two gaps, their focused associations with disease severity, organ dysfunction, and metabolic parameters, their prognostic value for three-month all-cause mortality, and candidate metabolic phenotypes defined by combining GapA-I with serum lactate.

### Description of baseline patient characteristics

Baseline characteristics of the study population are summarized in Table 1. Among the 140 included patients, 69 (49.3%) were non-survivors and 71 (50.7%) were survivors. No statistically significant differences were observed between survivors and non-survivors in terms of sex, age, height, weight, BMI, reason for ICU admission, or underlying comorbidities (including coronary heart disease, hypertension, and diabetes). Patients in the death group had a higher mean arterial pressure (MAP) at T0 than those in the alive group (86 ± 12 vs. 80 ± 12 mmHg, *p* = 0.002), whereas enteral nutrition use and parenteral nutrition use at T0 did not differ significantly between the two groups (*p* = 0.407 and *p* = 0.745, respectively).Additional clinical characteristics and illness-severity parameters stratified by survival status are presented in Table 2.

**Table 1:** Baseline characteristics of critically ill patients admitted to the intensive care unit

Variable	Survival status			*p* value^b^
	Total	Death	Alive	
	(*N* = 140)^a^	*N* = 69^a^	*N* = 71^a^	
Gender				0.868
Female	68 (48.57%)	33 (47.83%)	35 (49.30%)	
Male	72 (51.43%)	36 (52.17%)	36 (50.70%)	
Age (years)	66.00 [46.50, 76.00]	55.00 [47.00, 74.00]	68.00 [45.00, 76.00]	0.18
Height (m)	1.68 ± 0.05	1.67 ± 0.05	1.69 ± 0.05	0.077
Weight (kg)	66.67 [62.00, 71.94]	65.69 [62.00, 71.87]	68.00 [61.00, 72.00]	0.195
BMI (kg/m^2^)	23.81[22.53, 26.00]	23.72 [22.58, 26.06]	23.92 [22.49, 25.77]	0.195
Reason for ICU Admission				0.244
Sepsis	102 (72.86%)	46 (66.67%)	56 (78.87%)	
Autoimmune disease	17 (12.14%)	11 (15.94%)	6 (8.45%)	
Trauma	5 (3.57%)	4 (5.80%)	1 (1.41%)	
Cardiogenic shock	16 (11.43%)	8 (11.59%)	8 (11.27%)	
Coronary Heart Disease				0.678
No	111 (79.29%)	56 (81.16%)	55 (77.46%)	
Yes	29 (20.71%)	13 (18.84%)	16 (22.54%)	
Hypertension				0.348
No	101 (72.14%)	47 (68.12%)	54 (76.06%)	
Yes	39 (27.86%)	22 (31.88%)	17 (23.94%)	
Diabetes				0.068
No	109 (77.86%)	49 (71.01%)	60 (84.51%)	
Yes	31 (22.14%)	20 (28.99%)	11 (15.49%)	
MAP(mmHg)	83 ± 12	86 ± 12	80 ± 12	0.002
Enteral Nutrition	100 (71.4%)	52 (75.4%)	48 (67.6%)	0.407
Parenteral Nutrition	64 (45.7%)	33 (47.8%)	31 (43.7%)	0.745

a n (%).

b *p* values were calculated using Fisher’s exact test, Welch’s *t* test, or the Mann-Whitney U test, as appropriate.

**Table 2:** Comparison of baseline characteristics of critically ill patients stratified by survival

Variable	Survival status		*p* value^b^
	Death	Alive	
	*N* = 69^a^	*N* = 71^a^	
CRRT^d^			0.363
No	7 (10.14%)	4 (5.63%)	
Yes	62 (89.86%)	67 (94.37%)	
Vasoactive drugs^d^			0.736
No	29 (42.03%)	32 (45.07%)	
Yes	40 (57.97%)	39 (54.93%)	
Duration of mechanical ventilation (days)^c^	9.00 [6.00, 14.00]	16.00 [8.00, 22.00]	0.022
Duration of CRRT (days)^c^	13.00 [8.00, 26.00]	12.00 [7.00, 25.00]	0.609
Duration of vasoactive drug use (days)^c^	1.00 [0.00, 11.00]	2.00 [0.00, 14.00]	0.291
Duration of stay in ICU (days)	23.00 [10.00, 26.00]	41.00 [23.00, 73.00]	<0.001
APACHE II SCORE	17.00 [11.00, 22.00]	12.00 [6.00, 18.00]	0.001
SOFA SCORE	14.00 [10.00, 18.00]	9.00 [5.00, 13.00]	<0.001
PaO2_V_(mmHg)	46.30 [38.80, 55.30]	35.80 [32.10, 42.00]	<0.001
PaCO2_V_(mmHg)	34.80 [28.50, 41.60]	29.30 [26.90, 32.70]	0.005
HCO_3_^−^_V_(mmol/L)	18.40 [17.70, 20.00]	17.20 [16.40, 18.30]	0.015
Lac_V_(mmol/L)	1.30 [1.10, 1.60]	1.30 [0.90, 1.50]	0.448
PaO_2A_(mmHg)	111.00 [92.10, 141.70]	130.40 [100.40, 153.50]	0.088
PaCO_2A_(mmHg)	33.90 [29.80, 41.00]	30.60 [27.00, 36.00]	0.003
HCO_3_^−^_A_(mmol/L)	19.60 [18.50, 22.00]	19.00 [17.60, 20.70]	0.051
Lac _A_(mmol/L)	1.60 [1.20, 1.90]	1.40 [0.90, 1.90]	0.112
HR (beats/min)	89.09 ± 13.10	87.86 ± 14.31	0.597
RR (breaths/min)	15.00 [13.00, 17.00]	16.00 [15.00, 17.00]	0.508
T(°C)	37.09 ± 0.57	37.30 ± 0.65	0.051
SBP(mmHg)	126.33 ± 15.21	114.44 ± 17.96	<0.001
DBP(mmHg)	64.00 [59.00, 73.00]	60.00 [54.00, 70.00]	0.06

a n (%).

b *p* values were calculated using Fisher’s exact test, Welch’s *t* test, or the Mann-Whitney U test, as appropriate.

c The durations of mechanical ventilation, CRRT, and vasoactive drug use are expressed in days.

d The use of CRRT and vasoactive drugs represents utilization metrics within the ICU.

### Analysis of arterial-interstitial-venous glucose concentration differences

We next investigated whether glucose concentrations differed among arterial, interstitial, and venous compartments. Figure 1 shows the distribution of glucose concentrations across the three compartments. Arterial glucose (G_A_) was significantly higher than both interstitial glucose (G_I_) (10.97 ± 2.73 mmol/L vs. 10.37 ± 3.08 mmol/L; mean difference = 0.60 mmol/L, 95% CI: 0.31–0.90, *p* < 0.0001) and venous glucose (G_V_) (10.97 ± 2.73 mmol/L vs. 10.26 ± 2.40 mmol/L; mean difference = 0.71 mmol/L, 95% CI: 0.42–1.01, *p* < 0.0001). In contrast, no statistically significant difference was observed between G_I_ and G_V_ (mean difference = 0.11 mmol/L, *p* = 0.665). Given the minimal difference between G_I_ and G_V_, Gap_I-V_ was excluded from subsequent analyses, and further investigations focused on Gap_A-V_ and Gap_A-I_.

**Figure 1:**
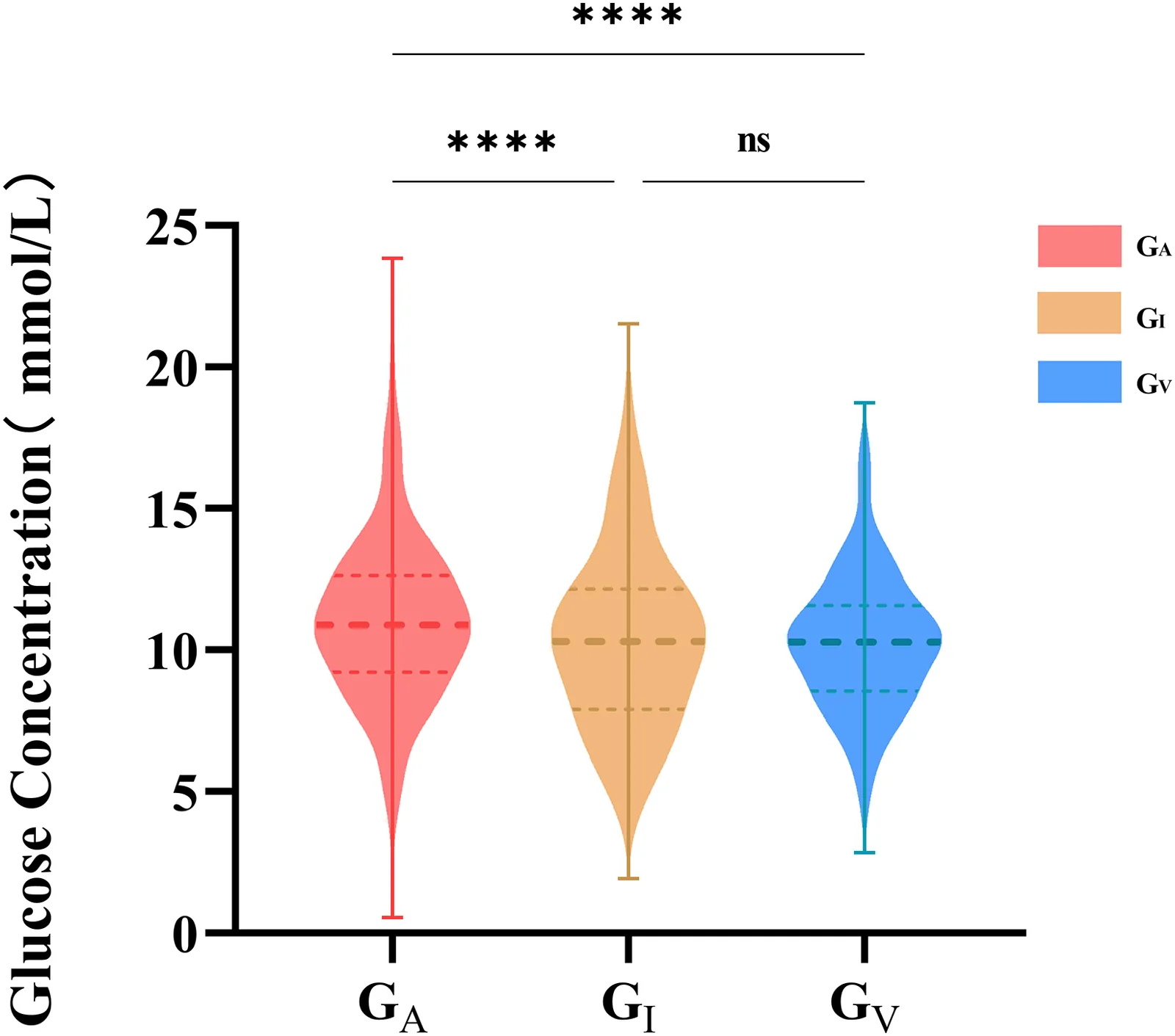
Comparative analysis of arterial, interstitial, and venous glucose concentrations in critically Ill patients Violin plots showing arterial glucose (G_A_), interstitial glucose (G_I_), and venous glucose (G_V_) concentrations in 140 mechanically ventilated critically ill patients. Violin plots show the data distribution, with dashed horizontal lines indicating the median and interquartile range (IQR). The vertical lines with caps indicate the minimum and maximum observed values. Comparisons were performed using one-way repeated-measures ANOVA followed by Tukey’s multiple comparisons test. ∗∗∗∗*p* < 0.0001; ns, not significant. G_A_ was significantly higher than both G_I_ and G_V_ (both *p* < 0.0001), whereas G_I_ and G_V_ did not differ significantly.

### Characteristics of glucose gap distributions

Although no statistically significant difference was observed between Gap_A-V_ and Gap_A-I_ (*p* = 0.3020) (Figure 2), both measures showed markedly different levels between survivors and non-survivors. Specifically, Gap_A-V_ was significantly higher in survivors than in non-survivors (1.07 [0.67, 1.52] vs. 0.27 [-0.02, 0.57], *p* < 0.001). Similarly, Gap_A-I_ levels were also substantially elevated in survivors compared to non-survivors (0.93 [0.20, 2.50] vs. 0.07 [-0.60, 0.65], *p* < 0.001).

**Figure 2:**
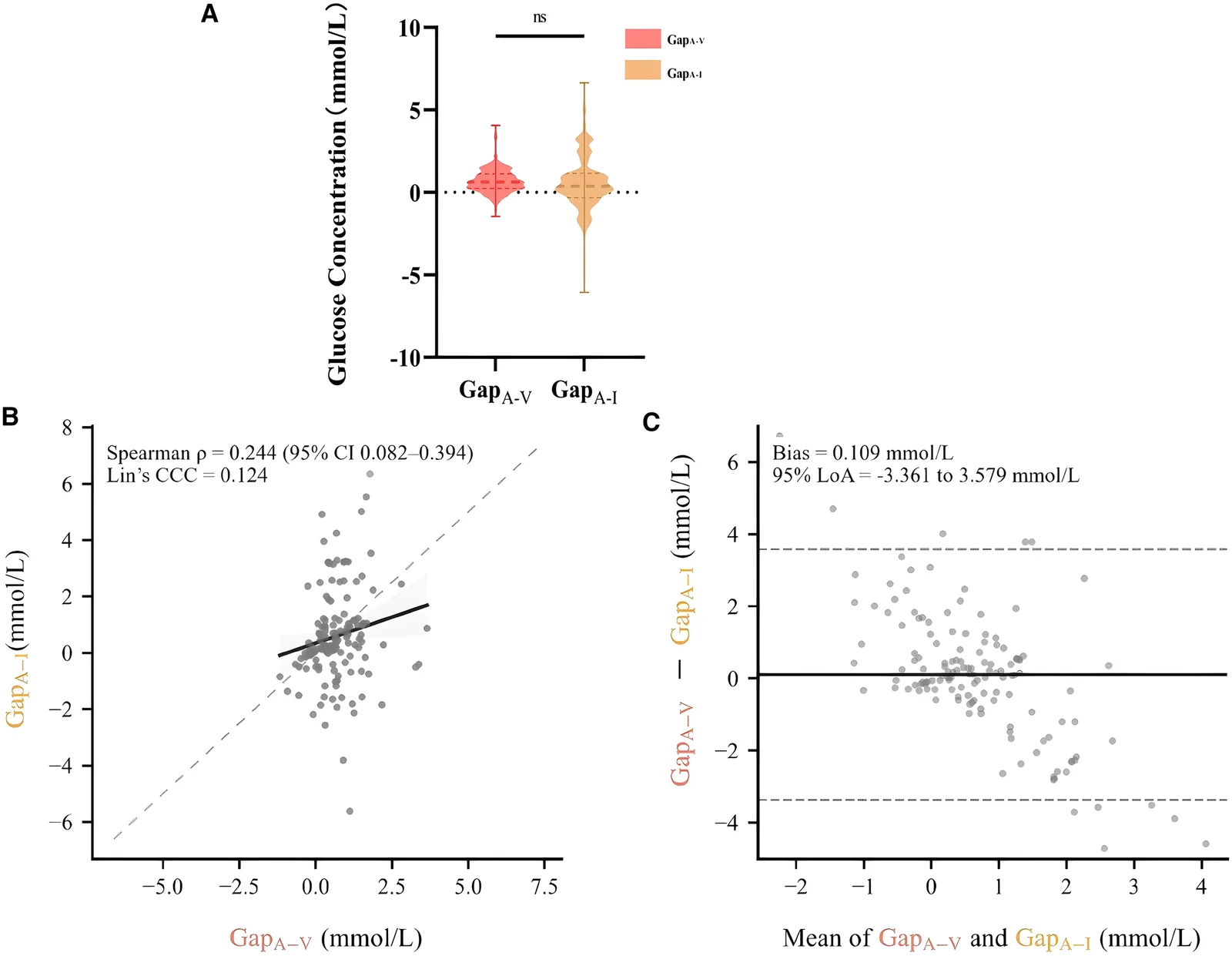
Distribution and agreement of Gap_A-V_ and Gap_A-I_ in critically ill patients (A) Violin plots compare Gap_A-V_ and Gap_A-I_ in 140 patients. Data are presented as median and interquartile range (IQR), with dashed horizontal lines indicating the median and quartiles. The vertical lines with caps indicate the minimum and maximum observed values. Comparisons were performed using the Wilcoxon matched-pairs signed-rank test; ns, not significant. (B) Spearman correlation between Gap_A-V_ and Gap_A-I_. The solid line represents linear regression; the gray shaded area represents the 95% CI; the dashed line represents the identity line. Spearman ρ = 0.244 (95% CI, 0.082–0.394); Lin’s concordance correlation coefficient (CCC) = 0.124. (C) Bland-Altman plot of Gap_A-V_ and Gap_A-I_. The solid line indicates the mean bias (0.109 mmol/L), and the dashed lines indicate the 95% limits of agreement (−3.361 to 3.579 mmol/L).

To further determine whether Gap_A-V_ could serve as a surrogate for Gap_A-I_, we assessed the direct association and agreement between the two measures. The correlation was weak (Spearman ρ = 0.244, 95% CI: 0.082–0.394), and concordance was limited (Lin’s concordance correlation coefficient = 0.124). Bland-Altman analysis revealed a small mean bias of 0.109 mmol/L but wide 95% limits of agreement (−3.361 to 3.579 mmol/L). These findings indicate substantial disagreement at the individual-patient level and do not support treating Gap_A-V_ as an interchangeable surrogate for Gap_A-I_ in this cohort.

### Correlations of the blood-interstitial glucose gaps with metabolic and disease severity indicators in critically ill patients

To investigate the clinical and metabolic relevance of the two glucose gaps, correlation analyses were performed with indicators of disease severity and metabolic status.

As shown in Figure 3, Gap_A-V_ showed significant positive correlations with PaO_2A-V_ (*r* = 0.32, *p* < 0.001), HCO_3_^−^_A-V_ (*r* = 0.31, *p* < 0.001), BMR(IC) (*r* = 0.27, *p* < 0.01), and VO_2_ (*r* = 0.27, *p* < 0.01), while demonstrating significant negative correlations with both APACHE II (*r* = −0.64, *p* < 0.001) and SOFA scores (*r* = −0.72, *p* < 0.001). Similarly, Gap_A-I_ showed strong correlations with metabolic parameters, exhibiting significant positive correlations with PaO_2A-V_ (*r* = 0.72, *p* < 0.001), VO_2_ (*r* = 0.78, *p* < 0.001), BMR(Harris-Benedict) (*r* = 0.23, *p* < 0.01), BMR(IC) (*r* = 0.79, *p* < 0.001), and VCO_2_ (*r* = 0.46, *p* < 0.001). Furthermore, Gap_A-I_ demonstrated significant negative correlations with PaCO_2A-V_ (*r* = −0.26, *p* < 0.01), HCO_3_^−^_A-V_ (*r* = −0.34, *p* < 0.001), APACHE II (*r* = −0.20, *p* < 0.05), and SOFA scores (*r* = −0.21, *p* < 0.05). Notably, Gap_A-I_ also correlated negatively with both arterial lactate (Lac_A_; *r* = −0.51, *p* < 0.001) and venous lactate (Lac_V_; *r* = −0.28, *p* < 0.001).

**Figure 3:**
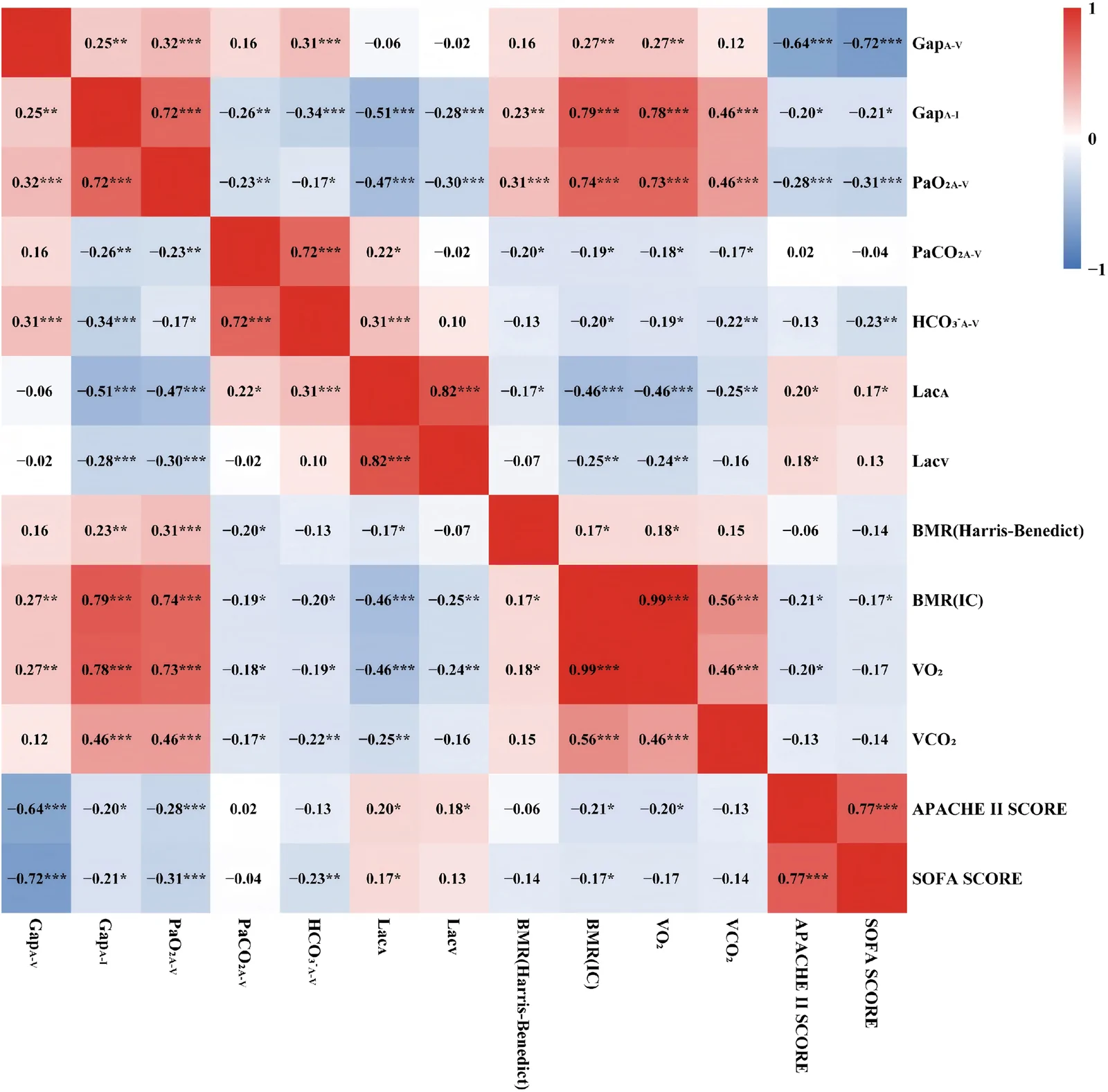
Correlations of Gap_A-V_ and Gap_A-I_ with Illness Severity and Metabolic Parameters in Critically Ill Patients Heatmap shows Spearman correlations of Gap_A-V_ and Gap_A-I_ with illness severity and metabolic parameters in 140 patients. Red indicates positive correlation and blue indicates negative correlation. The color intensity represents the correlation coefficient. Asterisks indicate unadjusted *p* values: ∗*p* < 0.05, ∗∗*p* < 0.01, and ∗∗∗*p* < 0.001. Benjamini-Hochberg false discovery rate (FDR)-adjusted results are provided in Table S2.

### Analysis of correlations between the blood-interstitial glucose gaps and markers of insulin resistance and thyroid hormones in critically ill patients

To further characterize the metabolic and endocrine associations of Gap_A-V_ and Gap_A-I_, we examined their relationships with insulin resistance indices and thyroid function parameters. As shown in Figure 4, Gap_A-V_ demonstrated negative correlations with fasting serum insulin (*r* = −0.38, *p* < 0.001) and the insulin resistance index (*r* = −0.22, *p* < 0.01), while showing significant positive correlations with FT4 (*r* = 0.62, *p* < 0.001) and TSH (*r* = 0.22, *p* < 0.05). In comparison, Gap_A-I_ exhibited strong negative correlations with both fasting serum insulin (*r* = −0.84, *p* < 0.001) and the insulin resistance index (*r* = −0.85, *p* < 0.001), along with a negative correlation with HbA1c (*r* = −0.45, *p* < 0.001). However, it showed a positive correlation with fasting serum C-peptide (*r* = 0.38, *p* < 0.001). Among the thyroid parameters examined, Gap_A-I_ showed a significant positive correlation exclusively with TSH (*r* = 0.41, *p* < 0.001), while exhibiting a negative correlation with A-TG (*r* = −0.19, *p* < 0.05).

**Figure 4:**
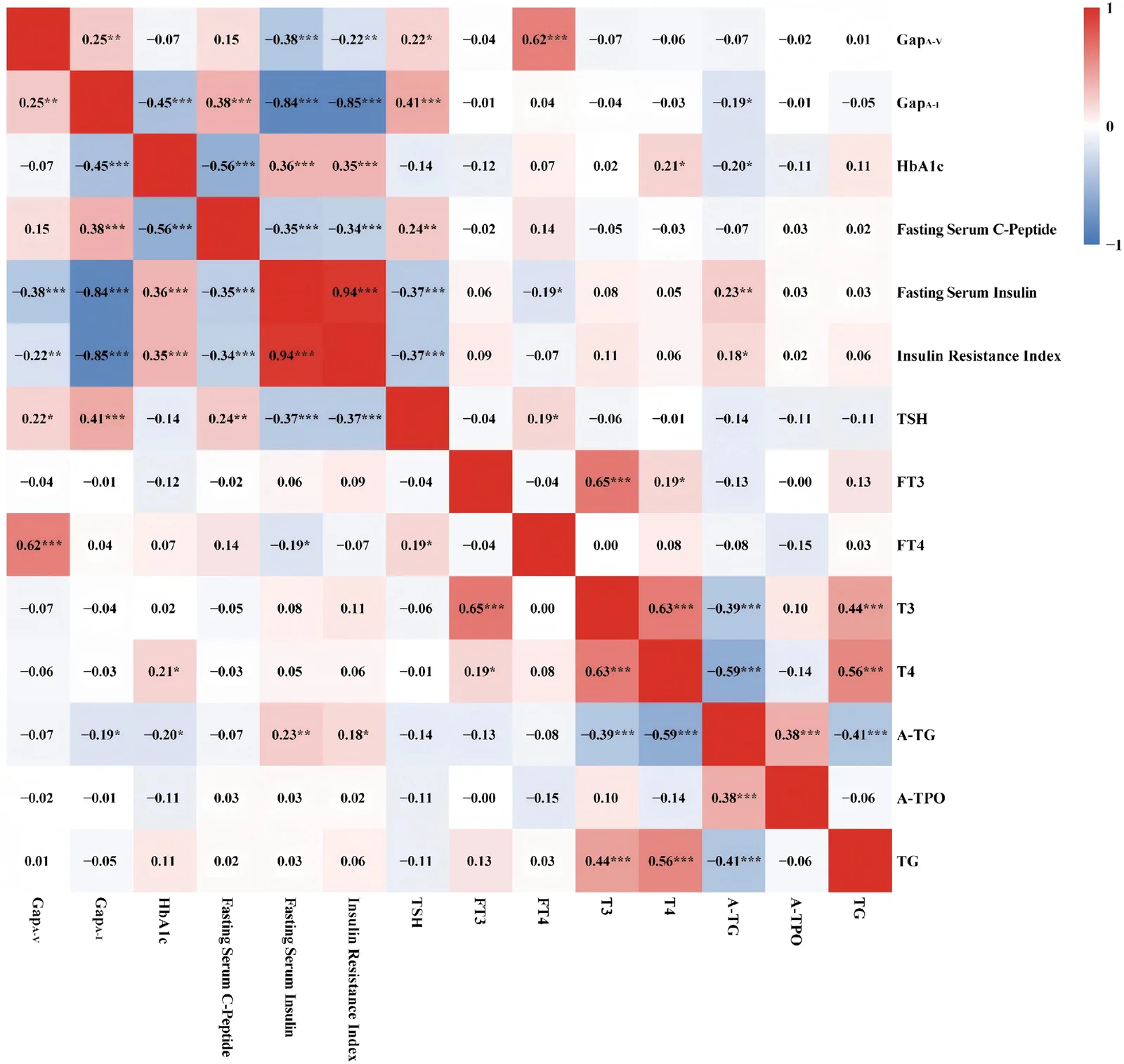
Correlations of Gap_A-V_ and Gap_A-I_ with insulin resistance-related parameters and thyroid function indices Heatmap shows Spearman correlations of Gap_A-V_ and Gap_A-I_ with insulin resistance-related parameters and thyroid function indices in 140 patients. Red indicates positive correlation and blue indicates negative correlation. Asterisks indicate unadjusted *p* values: ∗*p* < 0.05, ∗∗*p* < 0.01, and ∗∗∗*p* < 0.001. Benjamini-Hochberg FDR-adjusted results are provided in Table S2.

### Analysis of correlations between the blood-interstitial glucose gaps and organ dysfunction indicators in critically ill patients

To characterize the relationship between glucose gaps and specific organ dysfunction patterns, we extended the correlation analyses to individual biochemical indicators of organ injury, inflammation, nutritional status, and coagulation function. As shown in Figure 5, Gap_A-V_ demonstrated a significant positive correlation with WBC (*r* = 0.23, *p* < 0.01), while showing negative correlations with both ALT (*r* = −0.32, *p* < 0.001) and AST (*r* = −0.27, *p* < 0.01). It also exhibited significant positive correlations with creatinine (Cr; *r* = 0.20, *p* < 0.05) and blood urea nitrogen (BUN; *r* = 0.69, *p* < 0.001). Among nutritional indicators, Gap_A-V_ showed positive correlations with albumin (*r* = 0.27, *p* < 0.001), prealbumin (*r* = 0.19, *p* < 0.05), and transferrin (*r* = 0.18, *p* < 0.05). In terms of coagulation function, Gap_A-V_ demonstrated significant negative correlations with both prothrombin time (PT; *r* = −0.48, *p* < 0.001) and activated partial thromboplastin time (APTT; *r* = −0.36, *p* < 0.001).

**Figure 5:**
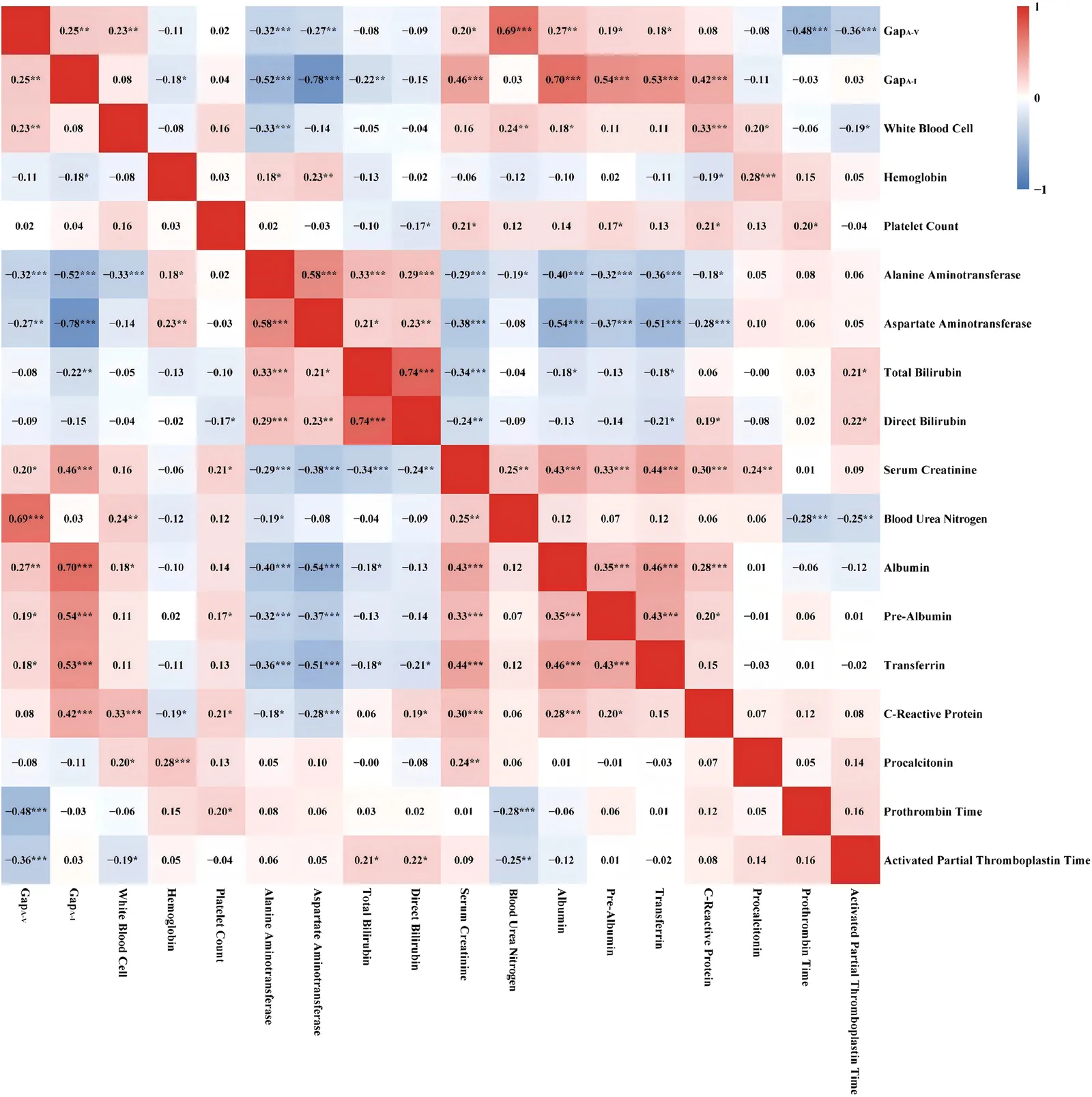
Correlations of Gap_A-I_ and Gap_A-V_ with Multiorgan Function Indices Heatmap showing Spearman correlations of Gap_A-V_ and Gap_A-I_ with multiorgan function indices in 140 patients. Red indicates positive correlation and blue indicates negative correlation. Asterisks indicate unadjusted *p* values: ∗*p* < 0.05, ∗∗*p* < 0.01, and ∗∗∗*p* < 0.001. Benjamini-Hochberg FDR-adjusted results are provided in Table S2.

On the other hand, Gap_A-I_ showed strong negative correlations with ALT (*r* = −0.52, *p* < 0.001), AST (*r* = −0.78, *p* < 0.001), and total bilirubin (TBIL; *r* = −0.22, *p* < 0.05). Additionally, Gap_A-I_ correlated positively with creatinine (Cr; *r* = 0.46, *p* < 0.001), albumin (Alb; *r* = 0.7, *p* < 0.001), prealbumin (Pre-Alb; *r* = 0.54, *p* < 0.001), and transferrin (Tf; *r* = 0.53, *p* < 0.001), while demonstrating a significant positive correlation with C-reactive protein (CRP; *r* = 0.42, *p* < 0.001). Gap_A-I_ also showed a negative correlation with hemoglobin (*r* = −0.18, *p* < 0.05).

To further compare the clinical and metabolic association profiles of Gap_A-I_ and Gap_A-V_, we evaluated differences in the absolute strengths of their Spearman correlations with individual clinical parameters using paired bootstrap analysis and false discovery rate correction. Gap_A-I_ showed stronger associations with several energy- and glucose-metabolism-related parameters, including resting energy expenditure, oxygen consumption, fasting insulin, and HOMA-IR. In contrast, Gap_A-V_ demonstrated stronger associations with several coagulation-related and selected physiological parameters. For several other variables, no meaningful differences in association strength were detected (Figure S2). These findings suggest that the two glucose gaps capture partially distinct clinical and metabolic information.

### Predictive value of the blood-interstitial glucose gap for prognosis in critically ill patients

Given the observed associations of glucose gaps with disease severity, metabolic characteristics, and organ dysfunction indicators, we next assessed their prognostic performance in predicting mortality among critically ill patients. As shown in Table 3 and Figure 6, the AUC of Gap_A-V_ was 0.863 (95% CI: 0.802–0.924), which was significantly higher than that of Gap_A-I_ (AUC = 0.708, 95% CI: 0.621–0.795; AUC difference = 0.155, 95% CI: 0.005–0.261, *p* = 0.004), indicating that both measures showed prognostic discrimination in critically ill patients. At the optimal cutoff values (Gap_A-V_: 0.66 mmol/L; Gap_A-I_: 0.275 mmol/L), Gap_A-V_ achieved 77.5% sensitivity and 82.6% specificity, while Gap_A-I_ showed 74.6% sensitivity and 66.7% specificity.

**Table 3:** Discriminatory power of blood-interstitial fluid glucose difference in predicting three-month all-cause mortality among critically Ill patients

Metric comparison	*p* value	AUC Difference(95%CI)
Gap_A-V_ - Gap_A-I_	0.004	0.155(0.005,0.261)

**Figure 6:**
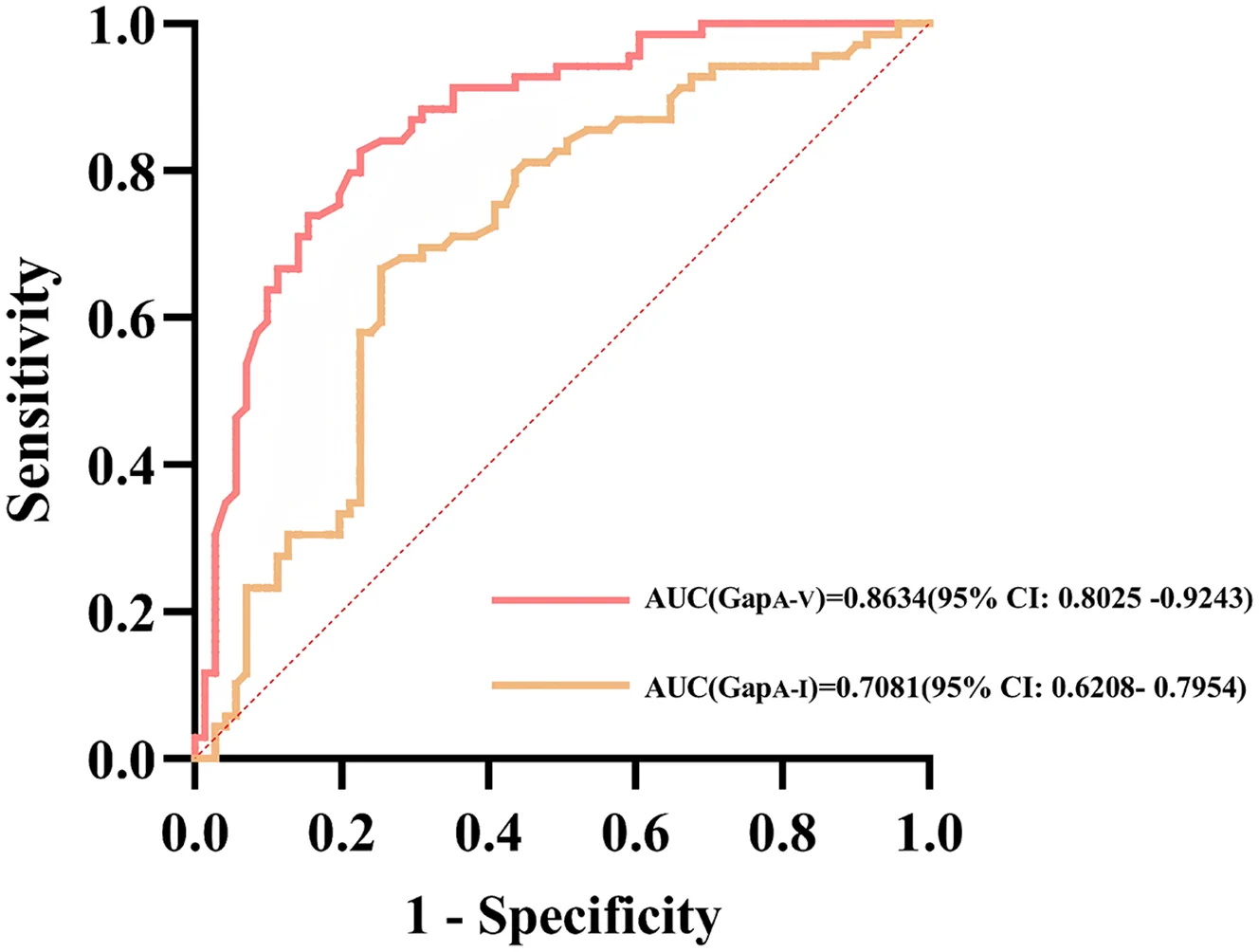
Discriminatory efficacy of blood-interstitial glucose gap for prognostic risk stratification in critically Ill patients: a receiver operating characteristic curve analysis Receiver operating characteristic (ROC) curves for Gap_A-V_ and Gap_A-I_ in predicting three-month all-cause mortality in 140 patients. The red dashed line is the reference line. Gap_A-V_: AUC = 0.8634 (95% CI, 0.8025–0.9243); optimal cutoff, 0.66 mmol/L; sensitivity, 77.5%; specificity, 82.6%. Gap_A-I_: AUC = 0.7081 (95% CI, 0.6208–0.7954); optimal cutoff, 0.275 mmol/L; sensitivity, 74.6%; specificity, 66.7%. See also Figure S1 and Table S3.

Bootstrap internal validation (1,000 resamples) showed that the discriminatory performance of Gap_A-I_ remained comparable to the apparent estimate, with a bootstrapped AUC 95% CI of 0.623–0.789 around an apparent AUC of 0.708. However, the bootstrapped 95% CI for the optimal cutoff value (0.275 mmol/L) ranged from 0.170 to 0.985 mmol/L, indicating that this threshold should be interpreted cautiously.

In patients without a prior history of diabetes (*n* = 109), the prognostic performance of Gap_A-V_ and Gap_A-I_ was consistent with that observed in the main analysis, with AUCs of 0.862 (95% CI, 0.794–0.930) and 0.680 (95% CI, 0.578–0.781), respectively. No statistically significant interaction was observed between diabetes status and either glucose-gap metric (Gap_A-V_ interaction *p* = 0.509; Gap_A-I_ interaction *p* = 0.660).

### Metabolic subtyping based on lactate and blood-interstitial glucose gap and its metabolic implications

The close associations between Gap_A-I_ and indices of insulin resistance and energy metabolism prompted us to investigate whether a combined assessment of Gap_A-I_ and serum lactate could identify distinct metabolic phenotypes in critically ill patients. Patients were stratified into four subtypes according to the optimal Gap_A-I_ cutoff (0.275 mmol/L) derived from ROC analysis and a clinically relevant lactate threshold (2.0 mmol/L): Normal (lactate <2.0 mmol/L and Gap_A-I_ ≥ 0.275 mmol/L), Utilization-Defective (lactate ≥2.0 mmol/L and Gap_A-I_ ≥ 0.275 mmol/L), Uptake-Defective (lactate <2.0 mmol/L and Gap_A-I_ < 0.275 mmol/L), and Dual-Defective (lactate ≥2.0 mmol/L and Gap_A-I_ < 0.275 mmol/L). The four subtypes comprised 66, 10, 43, and 21 patients, respectively.

To further evaluate whether these subtypes represented biologically distinct metabolic states, we compared insulin resistance and energy expenditure parameters across the four groups. As shown in Figure 7A, the metabolic phenotypes demonstrated a graded association with insulin resistance. The Normal subgroup exhibited the lowest insulin resistance index, whereas the Dual-Defective subgroup showed a significantly elevated index (*p* < 0.0001). Among the single-defect subgroups, the Uptake-Defective group had higher insulin resistance than the Utilization-Defective group; however, no statistically significant difference was observed between the Uptake-Defective and Dual-Defective subgroups.

**Figure 7:**
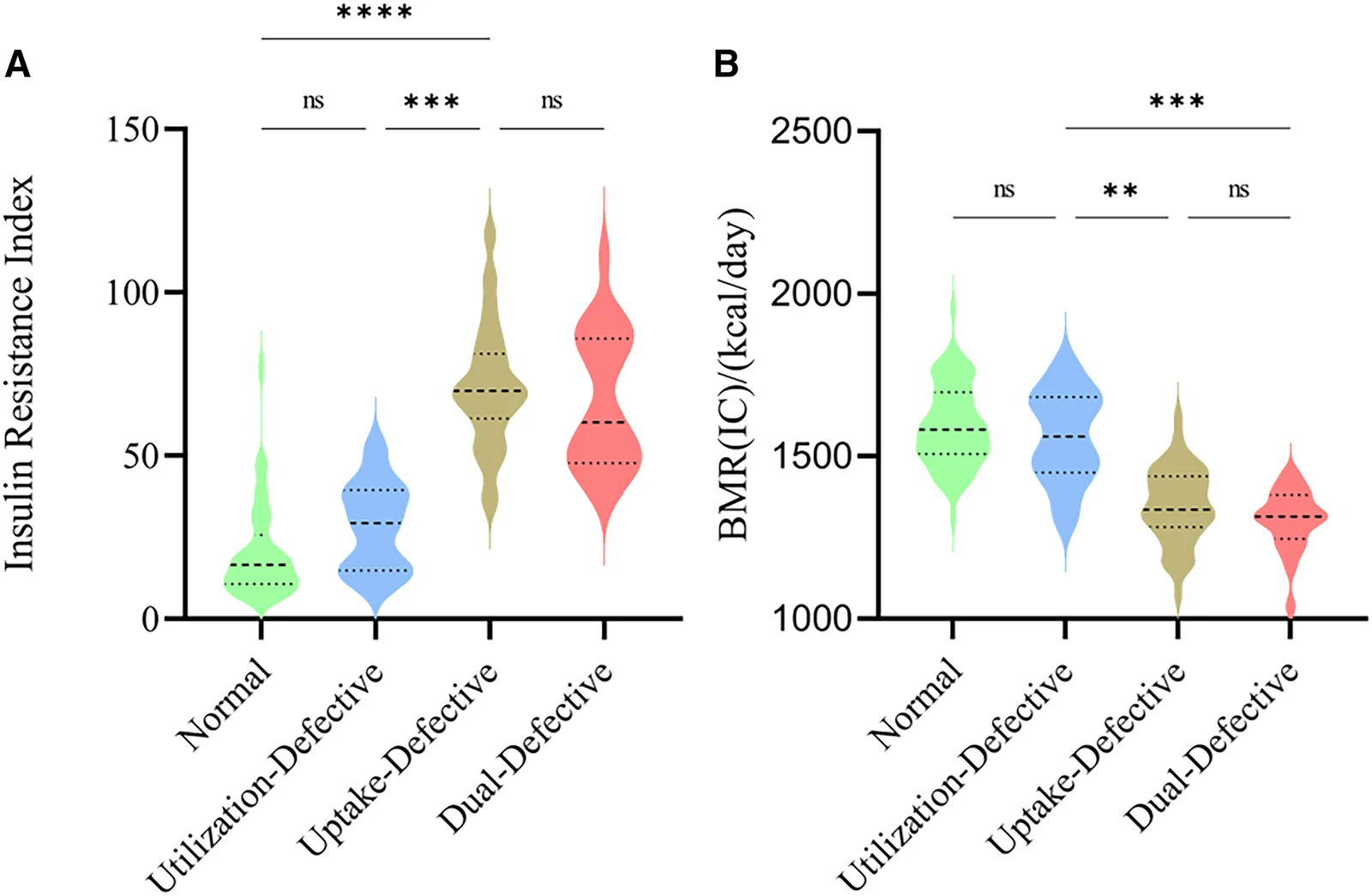
Comparative analysis of clinical parameters in patient stratification based on serum lactate and Gap_A-I_ cut-off values (A) Insulin resistance index and (B) basal metabolic rate measured by indirect calorimetry (BMR[IC]) across four metabolic phenotypes: normal (*n* = 66), utilization-defective (*n* = 10), uptake-defective (*n* = 43), and dual-defective (*n* = 21). Violin plots show the distribution; dashed lines indicate the median and IQR. Comparisons were performed using one-way ANOVA followed by Tukey’s multiple comparisons test. Horizontal lines indicate pairwise comparisons. ∗*p* < 0.05, ∗∗*p* < 0.01, ∗∗∗*p* < 0.001, and ∗∗∗∗*p* < 0.0001; ns, not significant. The comparisons shown are normal vs. utilization-defective, utilization-defective vs. uptake-defective, uptake-defective vs. dual-defective, and normal vs. dual-defective. See also Table S1.

Consistent with these findings, basal metabolic rate (BMR) also differed significantly among the metabolic subtypes (Figure 7B). The Normal subgroup displayed the highest BMR, whereas the Dual-Defective subgroup exhibited the lowest values (*p* < 0.0001). Both the Utilization-Defective and Uptake-Defective subgroups exhibited numerically lower BMR values than the Normal subgroup, although the difference between the Normal and Utilization-Defective subgroups was not statistically significant. Notably, the Utilization-Defective subgroup maintained a higher BMR than the Uptake-Defective subgroup (*p* < 0.05). Together, these findings indicate a graded pattern of metabolic impairment across the four phenotypes, with the Dual-Defective group exhibiting the most pronounced metabolic abnormalities.

To determine whether the metabolic phenotypes captured clinically meaningful differences beyond metabolic alterations, survival outcomes were subsequently compared among the four groups. The Utilization-Defective phenotype showed the highest survival rate (90%), which was comparable to that of the Normal phenotype (66.7%, *p* > 0.05). In contrast, survival rates were lower in the Uptake-Defective (27.9%) and Dual-Defective (28.6%) phenotypes, with no significant difference observed between these two groups (Figure 8).

**Figure 8:**
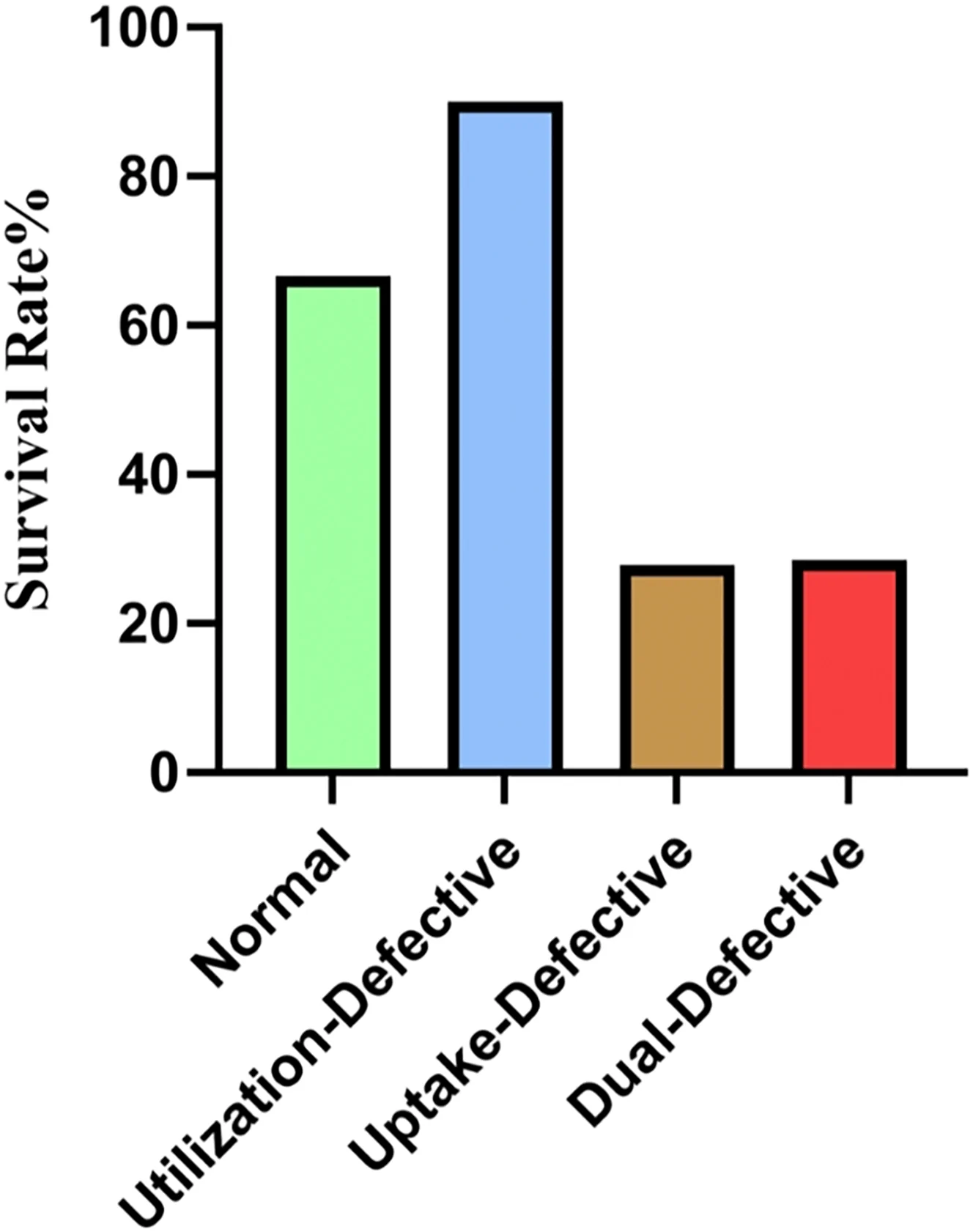
Comparative analysis of survival rate in patient stratification based on serum lactate and Gap_A-I_ cut-off values Bar graph shows three-month survival rates among four metabolic phenotypes: normal (*n* = 66; 66.7%), utilization-defective (*n* = 10; 90.0%), uptake-defective (*n* = 43; 27.9%), and dual-defective (*n* = 21; 28.6%). Data are presented as percentages. Survival rates were compared using the chi-square test or Fisher’s exact test, as appropriate. No statistically significant differences were observed between utilization-defective and normal (*p* > 0.05) or between uptake-defective and dual-defective (*p* > 0.05). See also Figure 7.

To further assess whether these survival differences were associated with baseline hemodynamic status or nutritional support, we compared relevant clinical variables among the four metabolic phenotypes. Mean arterial pressure (MAP), vasoactive drug use, enteral nutrition use, and parenteral nutrition use were comparable among the groups (overall *p* = 0.340, 0.880, 0.578, and 0.613, respectively; Table S1).

## Discussion

Our findings suggest that both Gap_A-V_ and Gap_A-I_ are associated with tissue metabolic status and correlate with parameters of energy metabolism, insulin sensitivity, and organ dysfunction in critically ill patients. For prognostic prediction, Gap_A-V_ exhibited superior performance compared to Gap_A-I_, while Gap_A-I_ showed slightly stronger correlations with energy metabolism parameters, insulin sensitivity, and multi-organ function indicators. The metabolic subtyping based on serum lactate and Gap_A-I_ revealed significant metabolic and prognostic differences among subtypes, further supporting the clinical value of these glucose gaps.

### Accuracy and rationale of the blood-interstitial glucose gaps

We found that G_A_ was significantly higher than both G_I_ (mean difference = 0.60 mmol/L) and G_V_ (mean difference = 0.71 mmol/L). Although no statistically significant difference was observed between G_I_ and G_V_, G_I_ tended to be higher than G_V_ (mean difference = 0.11 mmol/L). These findings are consistent with previous studies: Quinn et al.^20^ reported a significant difference between G_A_ and G_V_ (mean difference = 1.16 mmol/L); Rijkenberg et al.^21^ noted that G_A_ exceeded G_I_ (mean difference = 0.82 mmol/L); and Service et al.^22^ found a median absolute difference of 1.3 mmol/L between G_I_ and G_V_ in diabetic patients. From a physiological perspective, glucose is delivered to tissues through the arterial end of microcirculation, transported via interstitial fluid for cellular uptake and metabolism, with subsequent recovery of metabolites and unused glucose at the venous end. Since tissue cells take up glucose from the interstitial fluid rather than directly from capillary blood, G_I_ and G_V_ are theoretically expected to be comparable.^23^^,^^24^ Therefore, combining theoretical and empirical evidence, the non-significant difference between G_I_ and G_V_ suggests that Gap_I-V_ may lack clinical relevance and was consequently excluded from further analysis.

Notably, both Gap_A-V_ and Gap_A-I_ were significantly higher in survivors than in non-survivors, indicating their strong prognostic value: lower gap values were associated with higher mortality, with Gap_A-V_ demonstrating particularly superior predictive performance. Pathophysiologically, under normal capillary permeability, glucose passively diffuses from the vascular compartment to the interstitial fluid along the concentration gradient, followed by cellular uptake via facilitated diffusion mediated by glucose transporters (GLUTs).^25^ Our study also revealed that higher Gap_A-I_ values reflect preserved tissue glucose uptake and utilization capacity under stress conditions, preventing excessive glucose accumulation in the interstitium and indicating relatively intact microcirculatory perfusion and cellular metabolic function. Conversely, in severe infection or systemic inflammatory states, increased capillary permeability promotes extravasation of glucose-rich plasma components into the interstitial space. Additionally, insulin resistance, prevalent in non-survivors in our cohort, contributes to reduced gaps through dysfunction of the IRS-1/PI3K/Akt signaling pathway. This impairment may theoretically inhibit GLUT4 translocation to the cell membrane, compromise cellular glucose uptake, lead to glucose accumulation in the interstitial fluid, and ultimately result in a significant reduction or even negative values of the gaps.^26^ This suggests that insulin resistance may play a more prominent role than changes in vascular permeability in the reduction of glucose gaps in critically ill patients. Notably, the predictive value of Gap_A-I_ in our study differs slightly from Ma et al.'s findings in septic shock patients.^19^ The latter identified an optimal range for Gap_A-I_ (0.30–2.14 mmol/L) with the lowest mortality, while mortality increased outside this range. This discrepancy may be attributable to the fact that most Gap_A-I_ values in our cohort fell within 0.3–2.14 mmol/L, potentially leading to the observed trend of better prognosis with higher Gap_A-I_ values rather than identification of a safe range.

Although the pathophysiology of glucose gaps may differ between chronic diabetes and stress-induced dysglycemia in critical illness, the absence of significant interaction suggests that the main prognostic associations observed in this study were not solely driven by patients with pre-existing diabetes.

### The blood-interstitial glucose gap may reflect metabolic status in critically ill patients

Metabolically, this study observed that the blood-interstitial glucose concentration difference—particularly Gap_A-I_—demonstrated a significant positive correlation with BMR, oxygen consumption (VO_2_), and carbon dioxide production (VCO_2_) in critically ill patients. This association suggests that increased oxygen consumption might be accompanied by enhanced tissue glucose consumption and subsequent carbon dioxide generation. Consequently, Gap_A-I_ may serve as a surrogate marker for tissue glucose uptake, oxidative metabolism, and metabolite clearance, thereby potentially reflecting the tissue metabolic rate of critically ill patients.^27^

Furthermore, Gap_A-V_ showed a significant negative correlation with APACHE II and SOFA scores, while Gap_A-I_ was negatively correlated with lactate levels (Lac_A_, Lac_V_). These findings suggest that as disease severity and organ failure progress, impediments to tissue glucose uptake and utilization might become increasingly pronounced. It is generally postulated that such metabolic disturbances establish a vicious cycle, further exacerbating microcirculatory dysfunction and impairing mitochondrial substrate utilization.^28^ Notably, the negative correlation observed between Gap_A-I_ and both PaCO_2A-V_ and HCO_3_^−^_A-V_ further highlights its potential sensitivity in assessing overall metabolic and perfusion status. This suggests that Gap_A-I_ might function as an integrated metric synthesizing oxygen delivery, consumption, and tissue perfusion, offering potential clinical value in evaluating shock and guiding subsequent nutritional support and fluid resuscitation.

This study also observed a significant association between blood-interstitial glucose gaps and core endocrine metabolic indicators in critical illness: insulin resistance and thyroid hormone levels. Our results demonstrate a robust negative correlation between Gap_A-I_ and both fasting serum insulin and the insulin resistance index, suggesting that lower Gap values are associated with more severe insulin resistance. This phenomenon might be explained through the pathophysiology of critical illness: insulin resistance is the central link in glucose metabolic dysregulation. While insulin maintains glycemic homeostasis in health, impaired insulin-mediated glucose sensitivity leads to resistance and subsequent metabolic derangement in critically ill patients. Therefore, Gap_A-I_ may serve as an indirect reflection of this insulin-mediated metabolic disturbance.^29^ Consequently, our findings suggest that Gap_A-I_ has the potential to serve as a sensitive indicator for assessing the magnitude of insulin resistance in this pathological state.

Compared to existing methods, the Gap metric may offer a conceptually superior perspective. The hyperinsulinemic-euglycemic clamp, though the “gold standard”, is invasive, costly, and creates an artificial steady state that may fail to capture physiological glucose uptake dynamics.^30^ Similarly, simple indices such as HOMA-IR are mathematical estimates primarily reflecting hepatic insulin sensitivity rather than dynamic post-load tissue handling.^31^ While the oral glucose tolerance test (OGTT) is more physiological, it is confounded by factors such as insulin secretion capacity, making it difficult to isolate peripheral tissue sensitivity.^32^ In contrast, the Gap metric aims to directly and dynamically reflect the actual efficiency of local tissue glucose uptake. It theoretically circumvents confounders such as compensatory beta-cell secretion and integrates information regarding local microcirculatory perfusion and cellular metabolic status. When combined with lactate levels, this metric shows promise for refined metabolic subtyping. Thus, the Gap metric provides a potential novel vantage point for assessing tissue-level insulin resistance and metabolic function in critical illness.

Regarding thyroid function, critically ill patients often exhibit markedly reduced free thyroxine (FT4). Our finding that elevated Gap_A-V_ accompanies higher FT4 levels suggests that thyroid hormones might be a crucial compensatory factor in maintaining glucose metabolic capacity; higher FT4 levels may enhance the efficiency of peripheral tissue glucose uptake and utilization.^33^

It should be emphasized that the stronger correlations of Gap_A-I_ with metabolic parameters do not, by themselves, establish it as a superior metric. Gap_A-V_ can be obtained directly from routine blood gas analysis without a continuous glucose monitoring sensor and showed stronger prognostic discrimination for three-month mortality in the present cohort. We therefore regard Gap_A-V_ and Gap_A-I_ as complementary: Gap_A-V_ as a readily accessible prognostic indicator, and Gap_A-I_ as a more metabolically oriented metric whose use in phenotyping remains exploratory.

### The blood-interstitial glucose gap reflects organ function in critically ill patients

This investigation demonstrated significant associations between glucose gaps and organ function-related indicators in critically ill patients. Glucose metabolism is closely regulated to maintain energy supply and systemic homeostasis. The liver plays a central role in glucose metabolism through gluconeogenesis, glycogenolysis, glycolysis, and glycogen synthesis. In critical illness, impaired hepatic function may disrupt glucose handling and systemic glucose regulation.^34^

Renal dysfunction may also influence glucose compartment differences through alterations in tubular glucose handling and metabolic regulation. Therefore, changes in glucose gaps may reflect the complex interactions between metabolic disturbance and organ dysfunction during critical illness.^35^

Gap_A-V_ was associated with coagulation- and microcirculation-related indicators in our cohort. These findings suggest that arterial-venous glucose differences may capture aspects of systemic circulatory and metabolic alterations in critically ill patients. Considering the close relationship between endothelial injury, microcirculatory dysfunction, and impaired glucose transport during critical illness, further studies are needed to clarify the underlying mechanisms.^36^

Gap_A-I_ was associated with hepatocellular injury-related parameters and insulin resistance-related metabolic indices. These findings suggest that arterial-interstitial glucose differences may provide additional information regarding tissue metabolic alterations. However, interstitial glucose measurements may also be affected by peripheral perfusion abnormalities, capillary exchange alterations, and tissue edema, particularly in critically ill patients receiving vasopressors or experiencing fluid imbalance.^37^ Therefore, Gap_A-I_ should not be interpreted solely as a direct indicator of cellular glucose uptake, and the biological mechanisms underlying these associations require further investigation.

### Metabolic subtyping based on the blood-interstitial glucose gap: A potential approach for precision metabolic phenotyping

This study innovatively developed a metabolic subtyping model using serum lactate and Gap_A-I_, revealing distinct metabolic patterns and prognostic trajectories among critically ill patients. The Normal subgroup demonstrated the highest BMR (IC) and lowest insulin resistance index, accompanied by favorable outcomes, suggesting well-preserved glucose metabolism and effective metabolic-microcirculatory coupling. In contrast, the Dual-Defective subgroup exhibited pronounced abnormalities in the measured metabolic indices and a low survival rate (28.6%), comparable to that observed in the Uptake-Defective subgroup (27.9%).

Notably, patients in the “Utilization-Defective” subgroup exhibited the highest survival rate among all four subgroups. Based on their maintained Gap_A-I_ alongside elevated lactate, we speculate that these patients might still retain the capacity for rapid energy production through stress-induced accelerated glycolysis. Under stress conditions, even when insulin signaling pathway impairment restricts conventional glucose uptake routes, these patients might rapidly metabolize intracellular glucose via anaerobic glycolysis. Although metabolically inefficient, this “bypass” mechanism could provide crucial support for cellular survival during critical phases, thereby conferring a temporary survival advantage.^38^ However, it must be emphasized that this remains an exploratory hypothesis. Direct metabolic evidence, such as lactate/pyruvate ratios or mitochondrial respiration assays, was unavailable in our clinical cohort to definitively confirm this intracellular mechanism.

The Gap_A-I_ metric employed in our study demonstrates distinct advantages over single insulin resistance indices for assessing metabolic status. First, it offers greater economic feasibility and clinical practicality. Gap_A-I_ is derived from routinely monitored blood and interstitial glucose concentrations, requiring no complex calculations or additional expensive testing. More importantly, Gap_A-I_ provides an integrated physiological perspective: it reflects not only cellular glucose uptake but also encompasses the microcirculatory process of glucose delivery from vasculature to interstitial fluid. Thus, Gap_A-I_ represents a composite metric that simultaneously characterizes both “delivery” and “uptake” processes.

This integrative nature enables Gap_A-I_ to effectively complement lactate (representing oxygen supply-metabolic balance), together forming a precision metabolic phenotyping framework capable of distinguishing between “glucose utilization defects”, “glucose uptake defects”, or “dual defects”, a capability unattainable with single insulin resistance indices.

Additional subgroup analyses showed that the available baseline macro-hemodynamic variables, including mean arterial pressure (MAP), as well as the proportions of vasoactive drug use, enteral nutrition use, and parenteral nutrition use, did not differ significantly across the four metabolic phenotypes. These findings suggest that the Dual-Defective phenotype cannot be explained simply by overt differences in the available macro-hemodynamic variables or immediate nutritional-support patterns alone. Nevertheless, because detailed vasoactive-inotropic scores, cardiac output measurements, and microcirculatory monitoring data were unavailable, the present study cannot definitively distinguish a specific micro-metabolic abnormality from more complex hemodynamic impairment. Therefore, the interpretation of the Dual-Defective phenotype should remain cautious and hypothesis-generating.

In conclusion, admission glucose gaps were associated with disease severity, metabolic status, and prognosis in mechanically ventilated critically ill patients. Gap_A-V_ showed better prognostic performance, whereas Gap_A-I_ was more closely related to insulin resistance-associated features. Combined with lactate, Gap_A-I_ may also help identify distinct metabolic patterns with different clinical outcomes. These findings suggest that glucose-gap analysis may serve as a practical tool for early metabolic assessment in critical illness.

### Limitations of the study

This study has several limitations that should be acknowledged. First, as a single-center investigation with a relatively small sample size, the generalizability of our findings may be limited and requires further validation through multicenter, large-scale studies. Second, metabolic assessment relied solely on static glucose measurements at admission rather than continuous indirect calorimetry monitoring, potentially missing dynamic metabolic fluctuations throughout the day and introducing assessment bias. Future research should incorporate continuous monitoring methods to obtain more accurate metabolic trajectory data. Third, to ensure strict synchronization and comparability across arterial, venous, and interstitial glucose measurements for gap calculation, our metabolic assessment relied on a predefined single time point (T0, 6 h post-ICU admission) rather than 24-h average glucose or glycemic variability metrics. While this design improved cross-compartment comparability, it does not capture dynamic metabolic fluctuations over time. In addition, although T0 was predefined at 6 h after ICU admission to improve cross-compartment comparability and reduce the potential influence of early post-insertion instability, limitations related to sensor calibration and stabilization during the acute phase of critical illness cannot be excluded. Fourth, our mechanistic interpretations of the metabolic subgroups remain physiological inferences rather than direct intracellular evidence, while the optimal Gap_A-I_ cutoff (0.275 mmol/L) was both derived and applied within the same dataset. Bootstrap internal validation supported its discriminatory performance but the cutoff varied across resamples; accordingly, the metabolic subtyping findings should be regarded as exploratory and require external validation. Finally, the lack of significant differences in some indicators between survivors and non-survivors may partly reflect the limited temporal resolution of metabolic assessment. Measurements obtained at the predefined single time point (T0) may not capture peak values or dynamic changes in these parameters during disease progression, potentially limiting their prognostic discriminatory power.

## Resource availability

### Lead contact

Further information and requests for resources should be directed to and will be fulfilled by the lead contact, Wenkui Yu (yudrnj2@163.com).

### Materials availability

This study did not generate new unique reagents or materials.

### Data and code availability

• The individual-level clinical data generated and analyzed in this study are not publicly available due to patient confidentiality and institutional ethical restrictions. De-identified data may be made available from the lead contact upon reasonable request, subject to approval by the relevant institutional and ethics authorities.
• This paper does not report original code.
• Any additional information required to reanalyze the data reported in this paper is available from the lead contact upon reasonable request.

## Acknowledgments

This work was supported by the Prevention and Control of Emerging and Major Infectious Diseases-National Science and Technology Major Project (grant no. 2025ZD01902800).

## Author contributions

Z.Z. and T.G. conducted the study and drafted the main manuscript. X.L. (Xinran) conducted the study and performed data analysis. S.D. contributed to data collection and statistical analysis during the revision process. X.L. (Xiaohui), Y.X., X.J., S.C., and J.D. participated in data acquisition, analysis, or interpretation. M.C. prepared the charts and figures. G.W. provided important intellectual input during the revision process and contributed to manuscript revision and interpretation of the findings. W.Y. conceived and supervised the study, participated in study design and coordination, and critically revised the manuscript. All authors read and approved the final manuscript.

## Declaration of interests

The authors declare no competing interests.

## STAR★Methods

### Key resources table

**Table:** 

REAGENT or RESOURCE	SOURCE	IDENTIFIER
**Software and algorithms**		

R software (v4.3.1)	The R Foundation	https://www.r-project.org/
GraphPad Prism (v9.5)	GraphPad Software	https://www.graphpad.com/

**Other**		

Critically ill patients (Mechanically Ventilated)	Nanjing Drum Tower Hospital	N/A
Clinical trial registry	ClinicalTrials.gov	NCT07240103
Anytime CGM System (Subcutaneous sensor)	Jiangsu Yuyue Kailite	N/A
Stat Profile pHOx Ultra (Blood gas analyzer)	Nova Biomedical	N/A
GE Carescape R860 (Indirect Calorimetry)	GE Healthcare	N/A
HOMA-IR Calculation Formula	This paper (method details)	N/A

### Experimental model and study participant details

#### Study design and participants

This was a prospective observational study, registered at ClinicalTrials.gov (NCT07240103). We enrolled adult critically ill patients admitted to the intensive care unit (ICU) of Nanjing Drum Tower Hospital, The Affiliated Hospital of Nanjing University Medical School, between July 2023 and March 2024. The study was approved by the Ethics Committee of Nanjing Drum Tower Hospital (approval number: AF/SC-08/03.0), and written informed consent was obtained from all patients or their legally authorized representatives upon admission.

Inclusion criteria were: (1) provision of informed consent; (2) age >18 years; (3) receiving invasive mechanical ventilation upon admission; (4) anticipated ICU length of stay >24 h. Exclusion criteria included: (1) contraindications to subcutaneous glucose sensor placement (e.g., open abdomen, abdominal skin/soft tissue infection); (2) previous enrollment in this study during the current ICU admission; (3) concurrent participation in other interventional trials. All enrolled patients received standard care according to current critical care guidelines.

### Method details

#### Continuous glucose monitoring and synchronized sampling

All enrolled patients were equipped with subcutaneous glucose sensors (Anytime, Jiangsu Yuyue Kailite Biotechnology Co., Ltd., China) implanted in the left lower abdomen upon admission. The sites for continuous glucose monitoring and blood sampling were selected with practical considerations in mind, including ease of fixation and a lower risk of accidental dislodgement during routine ICU care and frequent bedside procedures. To allow for sensor equilibration and initial patient stabilization, the baseline for paired metabolic analysis (designated as T0 in this study) was established at 6 h post-ICU admission. At this time point, arterial and venous blood glucose concentrations were determined via blood gas analysis using samples drawn from the radial artery and median cubital vein of the non-infusion upper limb, respectively. Strictly synchronized with the blood sampling, interstitial fluid glucose (G_I_) levels were recorded using the glucose sensors.

#### Clinical and laboratory data collection

The blood-interstitial glucose gaps were subsequently calculated based on these simultaneous measurements. The following parameters were documented: Patient characteristics: demographic information, diagnoses, Sequential Organ Failure Assessment (SOFA) score, and Acute Physiology and Chronic Health Evaluation II (APACHE II) score. Metabolic parameters: arterial lactate (Lac_A_), venous lactate (Lac_V_), arterial–venous differences in carbon dioxide partial pressure (PaCO_2A-V_), oxygen partial pressure (PaO_2A-V_), and bicarbonate concentration (HCO_3_^−^_A-V_). All metabolic assays followed a predefined protocol performed by trained personnel. Blood gas analysis was conducted using the Stat Profile pHOx Ultra point-of-care analyzer (Nova Biomedical, Waltham, MA), calibrated every 8 h. Samples were confirmed free of air bubbles or clots, and automatic hematocrit correction was applied.Glucose metabolism markers: glycated hemoglobin (HbA1c), fasting serum C-peptide, fasting serum insulin, and insulin resistance index.Thyroid function: thyroid-stimulating hormone (TSH), free triiodothyronine (FT3), free thyroxine (FT4), triiodothyronine (T3), thyroxine (T4), anti-thyroglobulin antibody (A-TG), anti-thyroid peroxidase antibody (A-TPO), and thyroglobulin (TG).Organ function: alanine aminotransferase (ALT), aspartate aminotransferase (AST), total bilirubin (TBIL), direct bilirubin (DBIL), serum creatinine (Cr), and blood urea nitrogen (BUN).Nutritional status: albumin (Alb), prealbumin (Pre-Alb), and transferrin (Tf).Inflammatory markers: white blood cell count (WBC), C-reactive protein (CRP), and procalcitonin (PCT).Coagulation function: platelet count (PLT), prothrombin time (PT), international normalized ratio (INR), activated partial thromboplastin time (APTT), thrombin time (TT), fibrinogen (FIB), and D-dimer. Vital signs and clinical status (recorded concurrently at T0): respiratory rate (RR), heart rate (HR), temperature (T), systolic blood pressure (SBP), diastolic blood pressure (DBP), mean arterial pressure (MAP), enteral nutrition use, vasoactive drug use and parenteral nutrition use.

#### Calculation of blood-interstitial glucose gaps

The following glucose differences were computed:

$${{Gap}_{A-I}=G_{A}-G}_{I}$$

$${Gap}_{I-V}=G_{I}-G_{V}$$

$${Gap}_{A-V}=G_{A}-G_{V}$$

#### Assessment of resting energy expenditure

Basal metabolic rate (BMR) was determined at T0 using two approaches:

Harris–Benedict equation^39^:

Males: *BMR*(*kcal*/*day*) = 66.5 + 13.75 × *weight*(*kg*) + 5.003 × *height*(*cm*) - 6.775 × *age*(*years*).

Females: BMR(kcal/day)=655.1+9.563×weight(kg)+1.850×height(cm)−4.676×age(years)

Ventilator-based metabolic monitoring:

Resting energy expenditure (REE) was measured using the GE Carescape R860 ventilator equipped with an integrated metabolic module (GE Healthcare, USA). To ensure data reliability, a strict steady-state protocol was followed. After gas calibration, measurements were performed under sedation with the patient in a quiet, supine position, at least 30 min after any clinical interventions (e.g., suctioning, bathing) or ventilator adjustments. Each measurement session lasted for at least 30 min. The first 10 min were discarded to allow stabilization, and the subsequent 20 min were used for data collection. Steady state was predefined as a minimum of 5 consecutive minutes during which the variation in oxygen consumption (VO_2_) and carbon dioxide production (VCO_2_) remained within 10%. The values recorded during this steady-state period were averaged for analysis. Measurements failing to meet these criteria were excluded from the final analysis.^40^

#### Assessment of insulin resistance

Insulin resistance was assessed using the Homeostasis Model Assessment of Insulin Resistance (HOMA-IR) index. Based on the fasting samples collected at T0, the HOMA-IR value was calculated using the standard formula: ***HOMA***-***IR*** = (***FastingGlucose***[***mmol***/***L***] × ***FastingInsulin***[***μU***/***mL***])/22.5. Higher HOMA-IR values indicate a greater degree of insulin resistance.

#### Outcome measures

Patients were followed for three months, with the primary outcome being all-cause mortality during this period. Secondary outcomes included durations of organ dysfunction: vasopressor use, mechanical ventilation, and continuous renal replacement therapy (CRRT).

### Quantification and statistical analysis

Analyses were performed using R (v4.3.1) and GraphPad Prism (v9.5). Categorical variables are summarized as frequencies (percentages). Continuous variables were tested for normality using the Shapiro–Wilk test; normally distributed data are presented as mean ± SD and compared using the *t* test, whereas non-normally distributed data are presented as median [IQR] and compared using the Mann–Whitney U test. For comparisons across more than two groups, one-way ANOVA or the Kruskal–Wallis test was used, as appropriate, and categorical variables were compared using the chi-square test or Fisher’s exact test. For within-patient comparisons, arterial, interstitial, and venous glucose concentrations were analyzed using one-way repeated-measures ANOVA followed by Tukey’s multiple comparisons test. GapA-V and GapA-I were compared using the Wilcoxon matched-pairs signed-rank test. Correlations were assessed using Pearson or Spearman methods according to data distribution. Agreement between Gap_A-V_ and Gap_A-I_ was evaluated using Spearman’s rank correlation, with the 95% CI for Spearman’s ρ estimated using Fisher’s z transformation. Lin’s concordance correlation coefficient (CCC) was calculated to assess agreement. Bland–Altman analysis was used to evaluate bias and 95% limits of agreement, calculated as the mean difference ±1.96 SD. To compare the absolute strengths of Spearman correlations between Gap_A-V_ or Gap_A-I_ and each clinical or metabolic parameter, paired bootstrap resampling with 1,000 iterations was used to estimate Δ|*ρ*| = |*ρGap*_*A*-*I*_| - |*ρGap*_*A*-*V*_|, and 95% CIs were derived from the bootstrap distribution. To account for multiple testing in the correlation analyses shown in Figures 3, 4, and 5 and in the comparison of association strengths, *p* values were adjusted using the Benjamini–Hochberg false discovery rate (FDR) method; q < 0.05 was considered statistically significant. The predictive performance of the glucose gaps for three-month all-cause mortality was evaluated using receiver operating characteristic (ROC) curve analysis, and the area under the curve (AUC) was calculated to assess discriminatory ability. The optimal cutoff values were determined using the Youden Index (J), calculated as *J* = *sensitivity* + *specificity*- 1. The threshold corresponding to the maximum Youden Index was selected as the optimal cutoff. Additional subgroup analyses were performed in patients without a prior history of diabetes to evaluate the prognostic performance of Gap_A-V_ and Gap_A-I_ in the non-diabetic subgroup. Interaction terms between diabetes status and each glucose-gap metric were further tested to assess effect modification. To mitigate potential overfitting and optimism bias regarding the Gap_A-I_ cutoff used for metabolic subtyping, internal validation was performed using the bootstrap resampling method with 1,000 iterations to calculate 95% confidence intervals (CI) for the AUC and the cutoff. All tests were two-sided, and *p* < 0.05 was considered statistically significant.

### Additional resources

This study was registered at ClinicalTrials.gov under the identifier NCT07240103 (https://clinicaltrials.gov/study/NCT07240103).
