# Supplementary material for: Admission blood-interstitial glucose gap shows metabolic and prognostic associations in mechanically ventilated patients
Source: iScience. 2026 Nov 2;29(11):118093. doi: 10.1016/j.isci.2026.118093 (PMC13756104; doi:10.1016/j.isci.2026.118093)
Supplement: Supplementary file 1 [file mmc1.pdf]

iScience, Volume *29*

Supplemental information

Admission blood-interstitial glucose gap shows metabolic and prognostic associations in mechanically ventilated patients

Zhibo Zhang, Tao Gao, Xinran Liang, Sijia Dai, Xiaohui Liang, Yun Xu, Ming Chen, Xiang Ji, Shi Chen, Jianfeng Duan, Gang Wang, and Wenkui Yu

Supplementary Figure legend

Supplementary Fig.S1. Stability analysis of ROC-derived AUC and cutoff values for $Gap_{A\text{-}I}$ in critically ill patients.

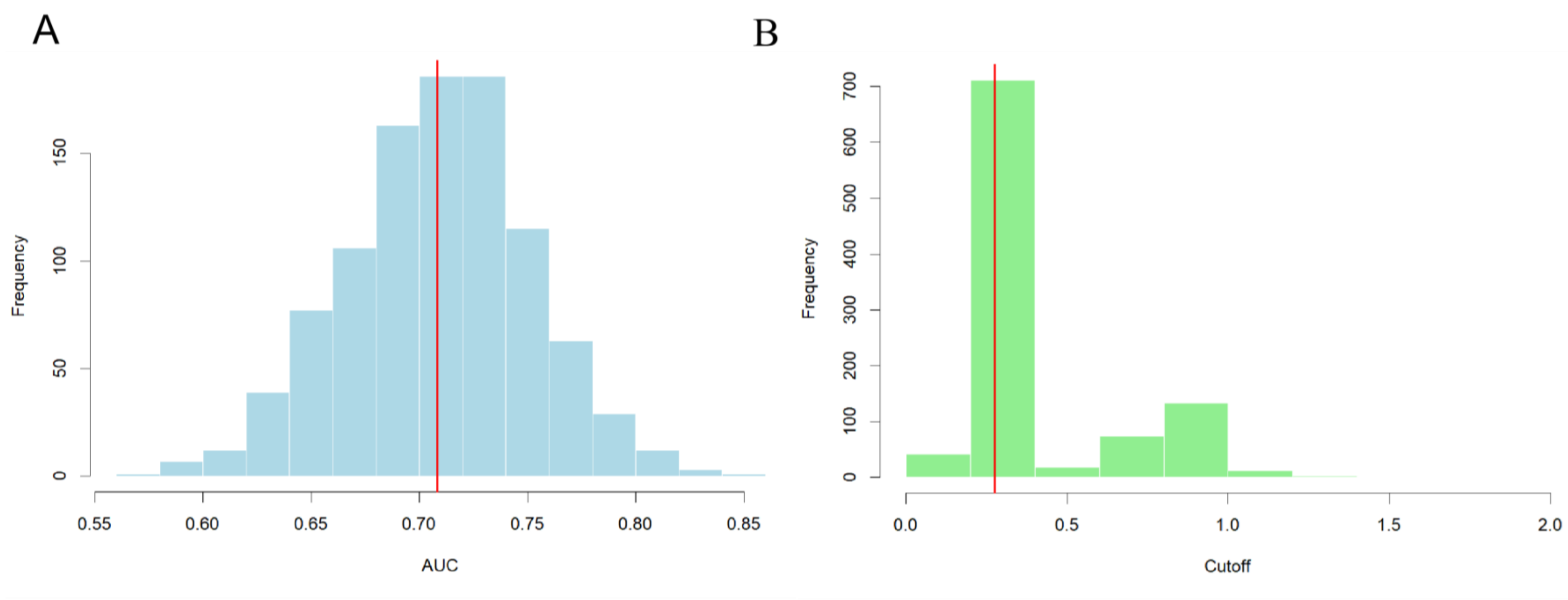

Bootstrap Distribution of AUC for $Gap_{A\text{-}I}$ Bootstrap Distribution of Youden Cutoff for $Gap_{A\text{-}I}$

(A) Bootstrap distribution of the area under the receiver operating characteristic curve (AUC) for $Gap_{A\text{-}I}$.

(B) Bootstrap distribution of the optimal GapA-I cutoff determined using the Youden index.

The red vertical lines indicate the corresponding estimates obtained from the original dataset.

Supplementary Fig. S2. Comparison of association strengths between Gap_{A-V} and Gap_{A-I} across clinical and metabolic parameters.

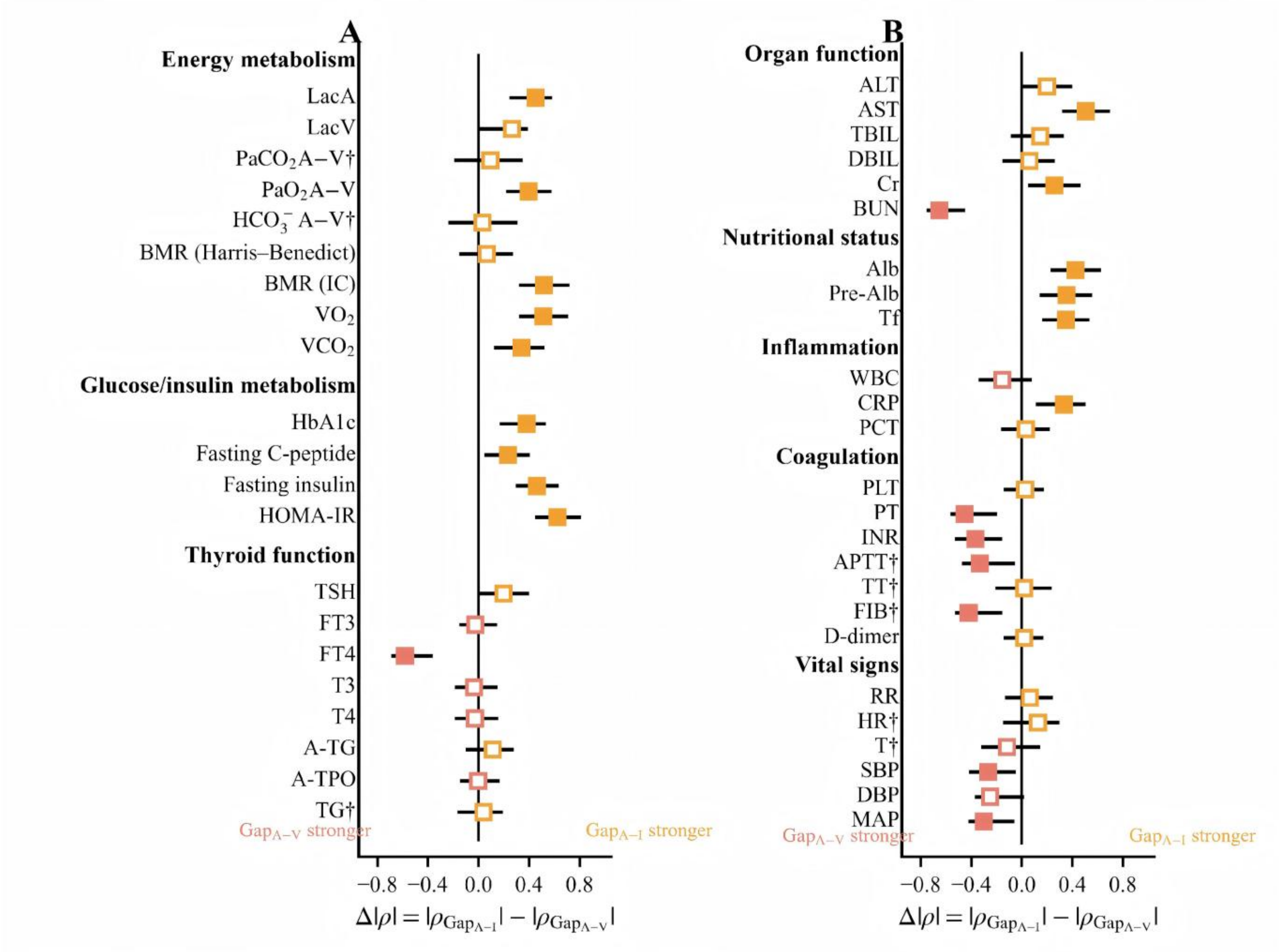

Filled: FDR $q < 0.05$; open: $q \geq 0.05$. † Opposite directions of association for Gap_{A-I} and Gap_{A-V}.

(A) Comparison of correlation strengths for metabolic and endocrine parameters.

(B) Comparison of correlation strengths for organ function, nutritional, inflammatory, coagulation, and vital-sign parameters.

Differences in absolute Spearman correlation coefficients were calculated as $\Delta|\rho| = |\rho(Gap_{A-I})| - |\rho(Gap_{A-V})|$. Positive values indicate stronger associations with Gap_{A-I}, whereas negative values indicate stronger associations with Gap_{A-V}. Filled squares indicate FDR-adjusted $q < 0.05$, while open squares indicate $q \geqslant 0.05$. The dagger symbol (†) denotes associations with opposite directions.

Supplementary Table legends

Supplementary Table S1. Mortality, hemodynamic characteristics, nutritional-support patterns, and crude odds ratios across metabolic phenotypes.

Metabolic phenotype	N	Death, n (%)	Alive, n (%)	MAP, mean ± SD	Vasoactive drug use, n (%)	Enteral nutrition use, n (%)	Parenteral nutrition use, n (%)	Crude OR for death (95% CI)
Normal	66	22 (33.3%)	44 (66.7%)	82.48 ± 11.85	35 (53%)	50 (75.8%)	27 (40.9%)	Reference
Utilization-Defective	10	1 (10%)	9 (90%)	78.37 ± 18.41	6 (60%)	8 (80%)	4 (40%)	0.22 (0.01–1.29)
Uptake-Defective	43	31 (72.1%)	12 (27.9%)	84.35 ± 12.82	26 (60.5%)	28 (65.1%)	23 (53.5%)	5.17 (2.28–12.34)
Dual-Defective	21	15 (71.4%)	6 (28.6%)	84.3 ± 9.65	12 (57.1%)	14 (66.7%)	10 (47.6%)	5.00 (1.77–15.71)
Overall P value		<0.001		0.340	0.880	0.578	0.613	

Abbreviations: MAP, mean arterial pressure; OR, odds ratio; CI, confidence interval. Metabolic phenotypes were defined according to lactate concentration and GapA-I. Data are presented as n (%), mean ± SD, or median [IQR], as appropriate. P values were calculated using appropriate univariable tests. Crude ORs for death were estimated by logistic regression using the Normal phenotype as the reference.

Supplementary Table S2. FDR-Significant Correlations of Gap_{A-V} and Gap_{A-I} with Clinical, Metabolic, and Organ Function Parameters.

	Gap_metric	Variable	Correlation	Raw *p*	FDR_q	Significant_after_FDR
Figure 3	Gap_{A-V}	PaO_{2A-V}	0.324	0.0000945	0.000167	Yes
Figure 3	Gap_{A-V}	$HCO_3^-{}_{A-V}$	0.308	0.00021	0.000346	Yes
Figure 3	Gap_{A-V}	BMR(IC/REE)	0.271	0.0012	0.00177	Yes
Figure 3	Gap_{A-V}	VO_2	0.27	0.00127	0.00178	Yes
Figure 3	Gap_{A-V}	APACHE II	-0.639	1.89E-17	8.71E-17	Yes
Figure 3	Gap_{A-V}	SOFA score	-0.717	2.36E-23	1.55E-22	Yes
Figure 3	Gap_{A-I}	PaO_{2A-V}	0.723	6.53E-24	5.01E-23	Yes
Figure 3	Gap_{A-I}	VO_2	0.78	6.67E-30	7.67E-29	Yes
Figure 3	Gap_{A-I}	BMR(Harris-Benedict)	0.225	0.00742	0.00948	Yes
Figure 3	Gap_{A-I}	BMR(IC/REE)	0.788	6.69E-31	1.03E-29	Yes
Figure 3	Gap_{A-I}	VCO_2	0.463	8.56E-09	2.32E-08	Yes
Figure 3	Gap_{A-I}	$PaCO_{2A-V}$	-0.256	0.0023	0.00312	Yes
Figure 3	Gap_{A-I}	$HCO_3^-{}_{A-V}$	-0.338	0.0000454	0.0000836	Yes
Figure 3	Gap_{A-I}	APACHE II	-0.205	0.0153	0.0172	Yes
Figure 3	Gap_{A-I}	SOFA score	-0.208	0.0137	0.0158	Yes
Figure 3	Gap_{A-I}	Arterial lactate	-0.508	1.42E-10	4.34E-10	Yes
Figure 3	Gap_{A-I}	Venous lactate	-0.282	0.000722	0.00115	Yes
Figure 4	Gap_{A-V}	Fasting serum insulin	-0.38	0.00000355	0.00000734	Yes
Figure 4	Gap_{A-V}	Insulin resistance index	-0.22	0.0091	0.011	Yes
Figure 4	Gap_{A-V}	FT4	0.619	3.48E-16	1.45E-15	Yes
Figure 4	Gap_{A-V}	TSH	0.215	0.0106	0.0126	Yes
Figure 4	Gap_{A-I}	Fasting serum insulin	-0.841	1.1E-38	2.53E-37	Yes
Figure 4	Gap_{A-I}	Insulin resistance index	-0.845	2.01E-39	9.24E-38	Yes
Figure 4	Gap_{A-I}	HbA1c	-0.45	0.000000024	5.82E-08	Yes
Figure 4	Gap_{A-I}	Fasting serum C-peptide	0.38	0.00000367	0.00000734	Yes
Figure 4	Gap_{A-I}	TSH	0.413	0.000000409	0.000000896	Yes
Figure 4	Gap_{A-I}	A-TG	-0.187	0.0268	0.028	Yes
Figure 5	Gap_{A-V}	WBC	0.235	0.00521	0.00685	Yes
Figure 5	Gap_{A-V}	ALT	-0.317	0.000133	0.000226	Yes
Figure 5	Gap_{A-V}	AST	-0.27	0.00128	0.00178	Yes
Figure 5	Gap_{A-V}	Creatinine	0.197	0.0198	0.0216	Yes
Figure 5	Gap_{A-V}	BUN	0.688	6.36E-21	3.25E-20	Yes
Figure 5	Gap_{A-V}	Albumin	0.274	0.00106	0.00162	Yes
Figure 5	Gap_{A-V}	Prealbumin	0.188	0.0264	0.028	Yes

Figure 5	Gap_{A-V}	Transferrin	0.176	0.0375	0.0375	Yes
Figure 5	Gap_{A-V}	Prothrombin time	-0.482	1.72E-09	4.94E-09	Yes
Figure 5	Gap_{A-V}	APTT	-0.359	0.0000129	0.0000247	Yes
Figure 5	Gap_{A-I}	ALT	-0.516	7.02E-11	2.31E-10	Yes
Figure 5	Gap_{A-I}	AST	-0.776	2.03E-29	1.87E-28	Yes
Figure 5	Gap_{A-I}	Total bilirubin	-0.223	0.00795	0.00989	Yes
Figure 5	Gap_{A-I}	Creatinine	0.456	0.000000015	3.83E-08	Yes
Figure 5	Gap_{A-I}	Albumin	0.697	1.23E-21	7.04E-21	Yes
Figure 5	Gap_{A-I}	Prealbumin	0.54	5.49E-12	2.1E-11	Yes
Figure 5	Gap_{A-I}	Transferrin	0.528	2.1E-11	7.43E-11	Yes
Figure 5	Gap_{A-I}	CRP	0.417	0.000000296	0.000000681	Yes
Figure 5	Gap_{A-I}	Hemoglobin	-0.179	0.0347	0.0354	Yes

Correlation analyses shown in Figures 3–5 were re-evaluated using the Benjamini–Hochberg false discovery rate method to account for multiple testing. The table presents the corresponding correlation coefficients, raw P values, FDR-adjusted q values, and significance after correction.

Supplementary Table S3. Diabetes Subgroup Analysis of the Prognostic Performance of Gap_{A-V} and Gap_{A-I}.

Metric	Non-diabetic subgroup (n)	AUC	95% CI	Interaction P value
Gap_{A-V}	109	0.862	0.794–0.930	0.509
Gap_{A-I}	109	0.680	0.578–0.781	0.660

Abbreviations: AUC, area under the curve; CI, confidence interval. The table summarizes the prognostic performance of Gap_{A-V} and Gap_{A-I} in patients without a prior history of diabetes (n = 109) and the corresponding interaction analyses by diabetes status.
